# Neurodevelopmental outcome in children between one and five years after persistent pulmonary hypertension of term and near-term newborns

**DOI:** 10.3389/fped.2024.1450916

**Published:** 2024-10-23

**Authors:** Laetitia Atlan, Lionel Berthomieu, Caroline Karsenty, Géraldine Gascoin, Catherine Arnaud, Sophie Breinig

**Affiliations:** ^1^ Pediatric and Neonatal Intensive Care Unit, University Hospital, Toulouse, France; ^2^ Neuropediatric Department, University Hospital, Toulouse, France; ^3^ Neonatal Intensive Care, University Hospital, Toulouse, France; ^4^ Centre for Epidemiology and Research in Population Health (CERPOP), UMR 1295, Study of Perinatal, Pediatric and Adolescent Health: Epidemiological Research and Evaluation Team, Toulouse III University, Toulouse University Hospital Center, Toulouse, France

**Keywords:** persistent pulmonary hypertension of the newborn, neurodevelopmental outcome, inhaled nitric oxide, ages and stages questionnaire ASQ-3, follow-up

## Abstract

**Background:**

Persistent pulmonary hypertension of the newborn (PPHN) is a serious condition that affects 1–2 per 1,000 newborns. Scientific data report the existence of neurological developmental abnormalities between 10 and 30%, but the description of these disorders linked with this situation of cerebral hypoxia and haemodynamic failure remains poorly documented.

**Objective:**

The main goal of this study was to describe the prevalence of neuro-psychomotor developmental disorders in children aged between one and five years old who have been hospitalised at birth in a neonatal intensive care unit for the management of PPHN.

**Methods:**

All of the newborns ≥34 weeks of gestational age (WGA) with PPHN, treated with inhaled nitric oxide in our neonatal intensive care unit between January 2015 and December 2019 were retrospectively enrolled. An ASQ-3 standardised questionnaire, adapted to the appropriate age (12, 24, 36, 48 and 60 months) was performed by the parents.

**Results:**

Fifty-five children (81% of answers) with a median age of 36 months (11–68), whose real age was close to the one of the questionnaire (12, 24, 36, 48 and 60 months), have been included in this study. There was 47% of pathological score [borderline: less than 1 standard deviation (SD) or suspect: less than 2SD] in at least one of the five studied domains, mainly in communication (25%) and individual and social skills (22%), despite a high overall score of 250 [220; 285] out of 300 that improved with age.

**Conclusion:**

This study showed a significant prevalence of neuro-psychomotor developmental disorders which justifies making more accessible a prolonged and adapted follow-up for early and multidisciplinary screening and management of these children with PPHN history. Larger cohorts are needed to better explore long term outcome of these vulnerable term neonates.

## Introduction

Persistent pulmonary hypertension of the newborn (PPHN) is a rare and serious although often reversible condition, subsequent to a failure in the extrauterine life adaptation occurring in 1–2 per 1,000 term or near term newborns (1, 2). The persistence of a fetal circulation via the two shunts of the ductus arteriosus (DA) and the foramen ovale (FO) induces a decrease in pulmonary and systemic blood flow, which is clinically responsible for desaturation and heart failure (3–6) with a significant risk of death ranging from 7% to 15% (7–9).

In the short run, PPHN therefore requires prompt and appropriate management in a neonatal intensive care unit (NICU) (10), which includes alveolar recruitment strategies if necessary (11), the validated (12) use of inhaled nitric oxide (iNO) since the end of the 90 s and hemodynamic support therapies, going as far as the use of inotropes and the set-up of an ECMO (8, 9) (extracorporeal membrane oxygenation).

With the improvement of physiopathological knowledge in PPHN and resuscitation techniques, the importance of the overall care of a developing child with the assurance of survival without comorbidities has gradually become a major issue.

These full-term or late preterm (13) delivered newborns present with greater cerebral vulnerability, a consequence of the pathology itself linked to prolonged cerebral hypoxia, the hemodynamic consequences of a decrease in the cerebral blood flow (14), the lack of maturity of the newborn central nervous system but also aggressive therapies.

Although current scientific data (15–22) report a prevalence rate of these neurodevelopmental disorders varying from 10% to 30% depending on the study, the neurological evolution's understanding remains poorly documented and significant differences stand undisputed between the populations (13, 20), the methods (8) and the results (23) studied. Some authors suggest that the presence of neurodevelopmental abnormalities is related to the initial severity of the disease (19), while others argue that the nature and the response of the treatment (24) influence the child's neuropathological development.

While most of the leading studies focused on the notion of major disability (9, 18) with cognitive, motor or sensory (16, 25) (auditory) sequelae, identified in small cohorts at one or two years, more recent data (17, 19, 26, 27) focus on the existence of minor developmental disorders such as more specific disorders of executive functions including memory, behaviour or language in these children during a more prolonged follow-up and off the radar of standard scores of neurodevelopment or intelligence. In 2010, Berti et al. (17) reported behavioural disorders in 26% and language disorders in 22%, without any pathological mental or motor score, of the 27 children followed for severe PPHN with an average age of 41 months.

In these developing children, sensitive to their environment, it seems essential to detect the early presence of these neuropsychomotor development disorders which may be the cause of a slight disability with a significant impact on school performance and an impaired quality of life. During this critical learning period of time with a high cerebral remodelling capacity, a long-term follow-up guarantees the screening and rehabilitation's efficiency as an established fact (28, 29).

The main goal of this study was to assess the prevalence and describe the intensity and characteristics of these neuropsychomotor development disorders in children with a history of PPHN, at kindergarten age from 1 to 5 years old, then, secondarily, to analyze the neonatal prognostic factors during the initial treatment that may affect long-term development.

## Methods

We performed an observational monocentric study based upon a survey with a retrospective analysis of neonatal data, named NEUROPHON study: NEURodevelopmental Outcome in children between one and five years after Persistent pulmonary Hypertension of the Newborn”. Over a period of five years, from January 1, 2015 to December 31, 2019, all newborns admitted and treated in the neonatal and paediatric intensive care unit of the Toulouse University Hospital (France) for PPHN were qualified for this study.

### Inclusion criteria

The inclusion criteria were based on a clinical and echocardiographic diagnosis of PPHN with the presence of labile desaturation in the neonatal period, the use of iNO, a gestational period of time ≥34 weeks of gestational age (WGA) and a chronological age below 28 days.

The exclusion criteria were the diagnosis of a genetic syndrome responsible for a delay in psychomotor development, the presence of a congenital defect such as diaphragmatic hernia or congenital heart disease, except for ventricular and atrial septal defects.

### Management

The care proceeding of these newborns was carried out in a tertiary center, considered as a reference center in the area for high-risk neonatal pathologies. This unit welcomes 980 patients per year and is the only one to perform neonatal ECMO in this geographic area. All the newborns included in this study, presenting with hypoxic respiratory distress, were treated with recent resuscitation techniques with conventional invasive or non-invasive adapted ventilation or by high frequency oscillatory (HFO) ventilation and all received iNO. The second-line therapies used in the unit were epoprostenol or treprostinil. Hemodynamic support with vasopressive and/or inotropic drugs was appropriately implemented according to echocardiographic evaluation.

#### Patients’ characteristics and data collection

Each patient's data were gathered retrospectively and analysed from the medical record for the perinatal period, the diagnosis and the evolution of PPHN.

Regarding the perinatal period, we collected data concerning the antenatal period on the pregnancy's follow-up, prenatal diagnosis of genetic syndromes or other associated pathologies and the maternal history as well as data concerning delivery and birth with sex, biometric parameters, term, adaptation to extrauterine life with the Apgar score and neonatal resuscitation in the delivery room.

Pertaining to the diagnosis, we have gathered the clinical, biological, paraclinical and in particular echocardiographic parameters on admission to our unit. These data were mainly related to the oxygenation of the newborn with the oxygenation index (OI) defined as “100 × FiO2 fraction of inspired oxygen × mean airway pressure/postductal aortic oxygen tension” [with an usual cutoff between 5 and 15 for mild hypoxemic respiratory failure (HRF), 25 or less for moderate, lower than 40 for severe, and above 40 for very severe] (30), ventilatory parameters, gas measurements. When we could not determine the OI, we used the saturation to assess the OSI (oxygen saturation index) (31). Haemodynamic parameters were collected, as well as echocardiographic parameters (direction of shunts in ductus arteriosus and foramen ovale, tricuspid regurgitation, right cavities dilatation, right and left ventricular function, pulmonary blood flow, sense of the septum curve, all parameters permitting to classify the patient in infra/iso/supra systemic pulmonary hypertension) during diagnostic confirmation. All newborns were classified for the underlying aetiology of PPHN according to the following causes: birth asphyxia, meconium aspiration syndrome (MAS), transient neonatal tachypnea (TNT), respiratory distress syndrome (RDS), neonatal sepsis and others.

As to the management of PPHN, we observed the ventilation variables (such as the ventilation duration and type, the oxygen supplementation duration FiO2 ≥90%), the administration of pulmonary vasodilators and the use of vasoactive drugs and inotropes as well as ECMO (type, duration). Concerning the hemodynamic aspect, the collected data made it possible to define a vasoactive-inotropic score (VIS) (32, 33). In order to assess the severity of PPHN in these children in a more objective way, for each hour of monitoring, the observation of one or more desaturation events (saturation ≤90%) or arterial hypotension (mean blood pressure MBP ≤35 mmHg) was counted once per hour to help define a cumulative score. Treatments associated with the management of PPHN such as surfactant, sedation and the use of antibiotic therapy have also been investigated. We also collected the occurrence of complications during hospitalization as well as the performance of a neurological assessment by imaging such as a cranial ultrasound, a computerized tomography (CT) scan, a magnetic resonance imaging (MRI) or an electroencephalogram (EEG), in case data were available.

### Follow-up programme ASQ

In order to study the psychomotor development in these surviving children treated for PPHN, we submitted a hetero-evaluation to the parents via a validated questionnaire to analyze the neuro-psychomotor development with a sensitivity of 86.1% and a specificity of 85.6% (34). This questionnaire is called Ages and Stages Questionnaires, third edition (ASQ-3) (35), the French version of which has been recommended (28, 36) to help caregivers identify and screen children's abilities and strengths.

This ASQ (37) is used routinely for the follow-up of premature babies (38–40) and post-anoxic encephalopathies (41). Each questionnaire is adapted to an age group targeting children from 1 to 66 months. Each questionnaire is presented in Supplementary Figure S1 (five documents in French language, one for each age). This is a screening tool which doesn't replace a physical evaluation by a specialist.

The ASQ-3 assesses the performance of cognitive and psychomotor development on five key points: communication, gross motor skills, fine motor skills, problem-solving, and individual/social skills. It is made up of six closed questions per area with three possible answers “Yes” (10 points), “Sometimes” (5 points) and “Not yet” (0 point), to establish a maximum total score of 300 points. To demonstrate the primary end-point, thanks to the total score obtained in each area, this screening tool allowed us to determine a classification of potential risks of disorders in psychomotor development according to three categories: normal, borderline, suspect. By comparing test results to established screening cutoff scores corresponding to 1 SD and 2 SD below the normative mean, the ASQ-3 enables distinguishing children to be monitored with cutoff scores, “borderline” between −1 SD and −2 SD presenting possible minor disorders, “suspect” children to be assessed with a score below −2 SD which could raise suspicion about an eventual developmental delay and require further evaluation.

After a telephone call to the parents, the test was emailed to all of them who had with children aged from 1 to 5 based on the corresponding age: 12, 24, 36, 48 and 60 months. Some of these children were already in a long-term follow-up programme within our medical network “Réseau Périnatalité Occitanie” (RPO), while others had never been evaluated by a remote hospitalisation specialist. It should be noted that parents were asked about their socioeconomic status, their children's level of education and whether they had medical or paramedical monitoring.

The primary outcome of this study was based on the prevalence of abnormal ASQs according to age and studied domains in our population.

### Ethical considerations

This study was led with the parents’ informed consent. In compliance with the 2012 *Jardé* law, the approval (number 2 021-A01019-32) of the patients’ committee (CPP: “comité de protection des personnes”) has been granted for this study on July 2, 2020.

### Statistical analysis

Using conventional methods, we performed a descriptive analysis of the data from the study population with the following appropriate indicators: for qualitative variables, numbers and percentages; for quantitative variables, either a position indicator and its dispersion index such as the mean and the standard deviation (±SD) in the case of normal distribution or the median with the interquartile range and/or rank [minimum-maximum] in the event of an asymmetric distribution.

Statistical tests were carried out using a two-sided approach with an alpha error risk of 5%. When we had a Gaussian distribution and all the prerequisite conditions are met, we used a *Student t-test*; otherwise, we chose a nonparametric *Wilcoxon Mann Whitney* test.

Qualitative data have been expressed as percentages with a 95% confidence interval (CI 95%). Qualitative variables have been compared using the *Chi-square test* (if the theoretical number is higher than 5) or *Fisher's exact test*, as appropriate. Quantitative data were compared using Pearson's or Spearman's correlation, based on statistical relevance. As for the secondary objectives, we considered a univariate analysis using the logistic regression model developed according to the results of the bivariate analysis and data from the literature, and in a second time a multivariate regression. Were included in the model head circumference at birth, birth weight, preductal minimal saturation, duration and dose of dobutamine, cord arterial pH, duration of sedation.

## Results

**Figure 1:**
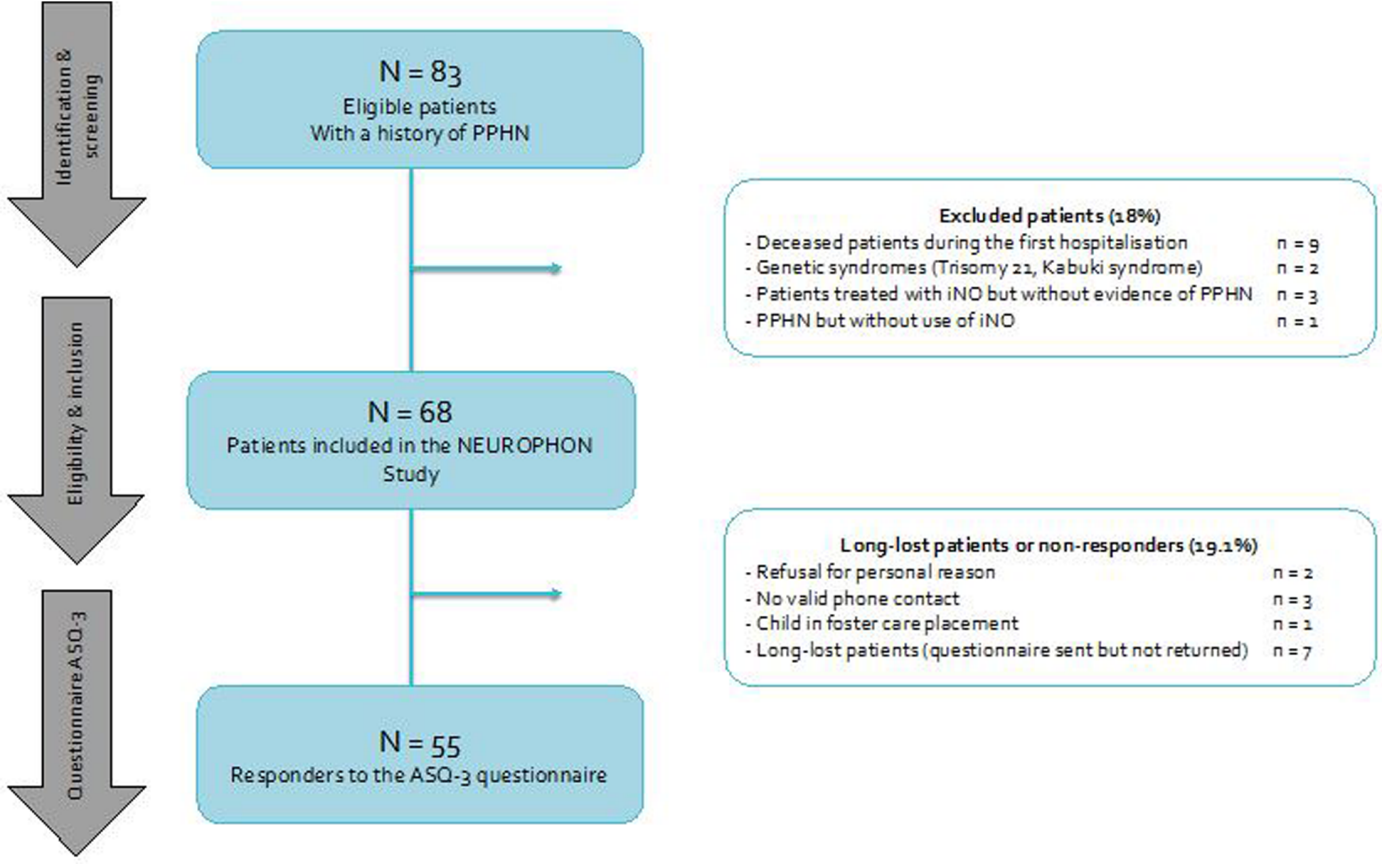
Study flow chart. PPHN, persistent pulmonary hypertension of the newborn; iNO, inhaled nitric oxygen; NEUROPHON study, NEURodevelopmental outcome in children between one and five years after | persistent pulmonary hypertension of the newborn.

Over the 2015–2019 period, 83 children treated for PPHN were identified. Nine (10.84%) out of fifteen excluded patients died during intensive care hospitalization, but none died after discharge. We included 68 children aged between 1 and 5 in the study to receive an ASQ-3 questionnaire adapted to their age. We obtained more than 80% (55 out of 68) of responses to the questionnaires. Figure 1 presents the flow chart. All the analyzed results relate to perinatal data and the current development of the 55 children who answered back.

### Population of the study

#### General data

The cohort of this study consisted of children with a predominance of male sex (56.4%), delivered at full-term on average at 39.1 (±2.19) WGA, via caesarian section (47.3%) in an emergency context (56.4%). The neonatal morphological characteristics were a mean birth weight of 3,152 g (±516), 20% of the participants were small for gestational age (SGA), with a weight <10th percentile). The data on postnatal adaptation found an Apgar score at 1 min of 5.9 (±3.7) and at five minutes of 6.9 (±3).

**Figure 2:**
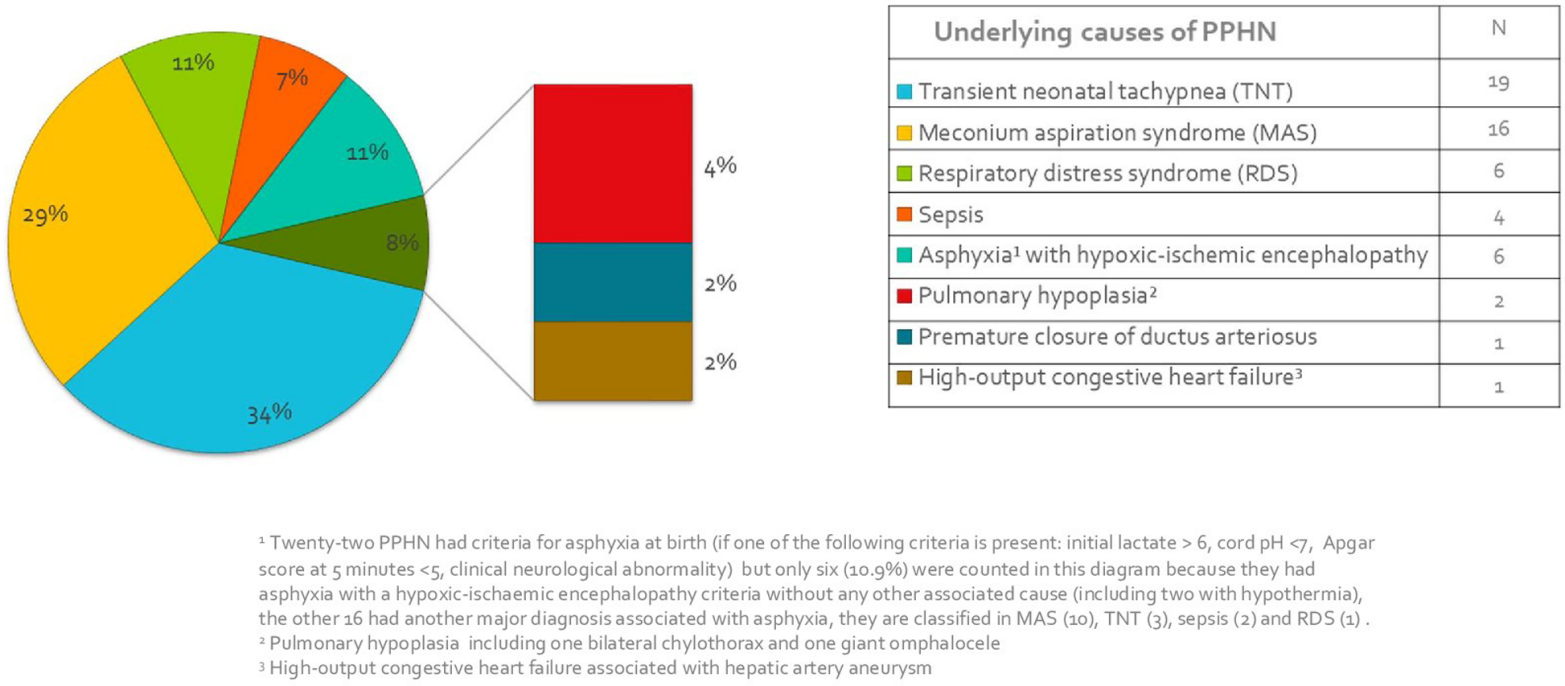
Distribution of aetiologies related to PPHN. PPHN, persistent pulmonary of the newborn.

Table 1 shows perinatal characteristics, clinical and paraclinical data post-admission to the ICU collected for the 55 children. The main causes of PPHN found were transient neonatal tachypnea (34.5%) and meconium aspiration syndrome (29.1%), all of the underlying aetiologies are listed in Figure 2.

**Table 1:** Characteristics of the population of the study, clinical, paraclinical informations and management of the PPHN.

	*N* = 55
Pre- and per-partum characteristics	
Outborn birth^a^, *n* (%)	45 (81.8%)
Rupture of membranes >12 h, *n* (%)	6 (10.9%)
Meconium amniotic fluid, *n* (%)	25 (45.5%)
Perinatal cardiac arrhythmias, *n* (%)	30 (54.5%)
Maternal fever, *n* (%)	14 (25.5%)
Chorioamnionitis, *n* (%)	2 (3.6%)
Neonatal characteristics	
Prematurity^b^, *n* (%)	10 (18.2%)
Post-term pregnancy^c^, *n* (%)	4 (7.3%)
Neonatal resuscitation in the delivery room^d^, *n* (%)	47 (85.5%)
Lactate Umbilical-cord blood gas (mmol/L) (*n* = 48), mean (±SD)	6.7 (3.8)
Clinical and paraclinical data	
Haemodynamic data	
Minimal Mean Blood Pressure (MBP, mmHg), mean (SD)	30.9 (5.8)
Hypotension cumulative score^e^, median [IQR]	2 [0; 5]
Haemodynamic shock, *n* (%)	37 (67.3%)
Oxygenation and ventilation data	
Minimal pre-ductal saturation (%), mean (SD)	79 (12)
Desaturation cumulative score^e^, median [IQR]	4 [1; 10]
Ventilation parameters: total duration of maximal FiO2^f^, median [IQR]	14 [4; 66]
Chest radiograph with hyperlucency or without opacities, *n* (%)	52 (94.5%)
Neurologic data	
Pathological Sarnat score (2 or 3), *n* (%)	4 (7.3%)
Intensive care management data	
Total length of hospital stay (days), median [IQR]	17 [11.5; 30.5]
Ventilation	
Delay of intubation from birth (minutes), median [IQR]	120 [22.5; 140.0]
Usage of high frequency oscillation, *n* (%)	29 (53.7%)
Surfactant instillation (one ou two doses), *n* (%)	33 (60.0%)
Haemodynamic	
Fluid resuscitation, *n* (%)	33 (60.0%)
Total duration of inotropic-vasopressive drugs (days), mean (SD)	3.2 (2.9)
VIS^g^, median [IQR]	20 [10; 90]
Sedation and curarisation	
Duration of sedation (days), median [IQR]	4 [3; 8]
Curarisation, *n* (%)	26 (47.3%)
Therapeutic data specific to PPHN	
Initiation of iNO (delay from birth, in hours), median [IQR]	11 [6; 25]
Maximal dose of iNO (ppm), mean (SD)	16.3 (6.1)
Prostaglandin E1 (Alprostadil), *n* (%)	9 (16.4%)
Corticosteroids, *n* (%)	27 (49.1%)
Complications	
Early onset neonatal infection^h^, *n* (%)	7 (12.7%)
Secondary infection^i^, *n* (%)	4 (7.3%)
Blood transfusion, *n* (%)	21 (38.2%)
Methemoglobinemia (>3%), *n* (%)	1 (1.8%)
Prolonged nutritional support^j^, *n* (%)	25 (45.5%)

^a^ Outborn birth (outside our main hospital in Toulouse).

^b^ Prematurity: birth between 34^0/6^ and 36^6/6^ amenorrhea weeks (AW).

^c^ Post-term: birth after 42 AW (≥42^0/6^ AW).

^d^ Neonatal resuscitation in the delivery room means at least mask ventilation and included chest compression if necessary.

^e^ Hypotension or desaturation cumulative scores: Recording once per hour of one or more desaturation (≤90%) or arterial hypotension (MBP ≤35 mmHg).

^f^ Total duration of maximal FiO2 corresponding to the number of hours with Fraction of inspired oxygen ≥90%.

^g^ VIS: vasoactive-inotropic score, ≥20 high score and <20 low score.

^h^ Early onset infection was defined as occurring within the first 72 h of life.

^i^ Secondary infection was defined as a sepsis acquired later than the first 72 h of life.

^j^ Prolonged nutritional support by parenteral nutrition or nasogastric tube ≥8 days.

#### Respiratory and hemodynamic data

In 44 newborns, we calculated an oxygenation index of 22 [17.7; 29.25] with a minimum of 5.6 and a maximum of 100. The echocardiographic data carried out in all these newborns allowed us to determine a certain profile and an evolution of PPHN: in 67.3% of the babies, the echocardiography diagnosis was confirmed as of the admission into the unit, in a third, it appeared during the evolution. Most of them were labelled iso-systemic PPHN (50.9%), but in 15 children, a secondary worsening was observed, often leading to a supra-systemic PPHN (41.8%). Right and/or left ventricular failure was present in 34.5% of children (18.2% and 21.8%, respectively).

#### Management data

Regarding the care data, these newborns were hospitalized for 8 [6; 11.5] days in ICU and all received iNO during their stay for 63 [34; 125] hours. A second therapeutic line of pulmonary vasodilators was necessary in seven patients. Two patients were implanted with ECMO (one veno-venous VV and one veno-arterial VA ECMO) on the second day of life for a period of 5 days. Almost all of these newborns (98.18%) were intubated and ventilated for a period of 135 [96; 210] hours with a withdrawal on day 8 [5; 14]. Around 72.7% (*n* = 40) of them needed amine support, mainly with the use of vasopressor like dopamine (*n* = 32, 58.2%) or inotropic drugs like dobutamine (*n* = 22, 40%).

#### Neurologic data

When it came to neurological examination, 43 subjects (78.2%) did not present any abnormalities on discharge from hospitalization, 12 (22.8%) had a pathological examination with a tonus abnormality alone (5.5%) or associated with a vicious attitude or abnormal contact (16.4%). Five newborns presented with clinical or subclinical convulsions (objectified by an amplitude-integrated EEG or an EEG) and received a loading dose of anti-convulsant (Phenobarbital); two had an indication for a long-term anti-epileptic treatment (Levetiracetam). Nearly 50% (*n* = 27, 49.1%) of the population received a medical imaging examination by CT scan (5.5%), MRI (23.6%) or head ultrasound (29%), the results of which were normal for 17 of them (62.9%), found minor anomalies in 6 (22.2%) and major anomalies in 4 (14.81%) defined as hemorrhagic or ischaemic lesions. In our cohort, only two children with anoxic ischaemic encephalopathy underwent therapeutic hypothermia, but the treatment was stopped early in one of them due to complications from PPHN.

### ASQ-3: description of the results

#### Primary objective

**Figure 3:**
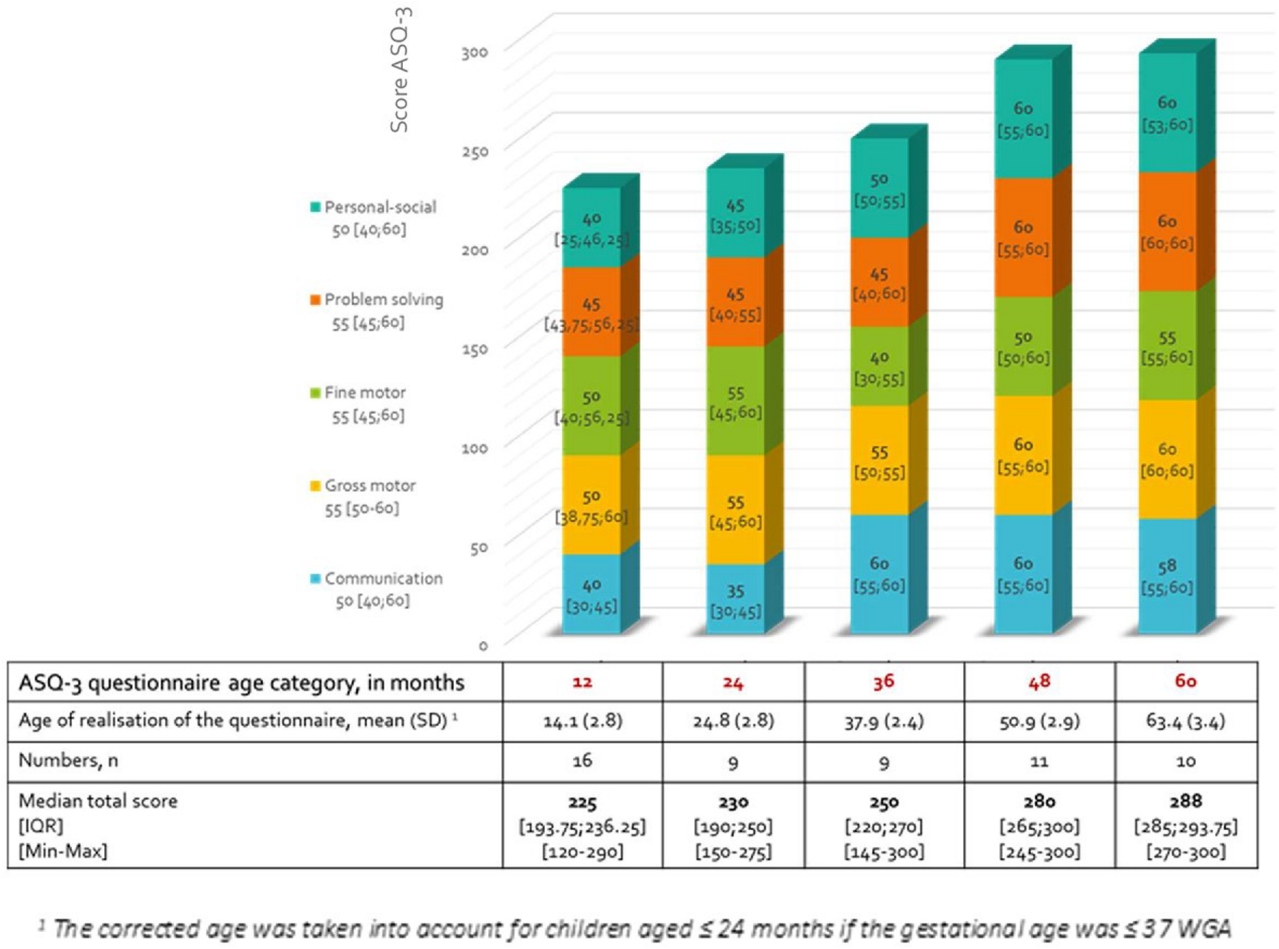
Results of median scores and interquartile range [IQR] of the ASQ-3 questionnaires in the five different domains according to the age category. Non-Gaussian distribution/ASQ-e: Ages and stages -3/WGA, weeks of gestational age.

The total median score was 250 [220; 285] (rank: 120–300) out of 300 in these 55 children with an average age of completing the questionnaire of 36 months (±18.6). As shown in Figure 3, the total score increase with years and differ according to the age of realization of the questionnaire, the latter being close to the children's real age.

**Figure 4:**
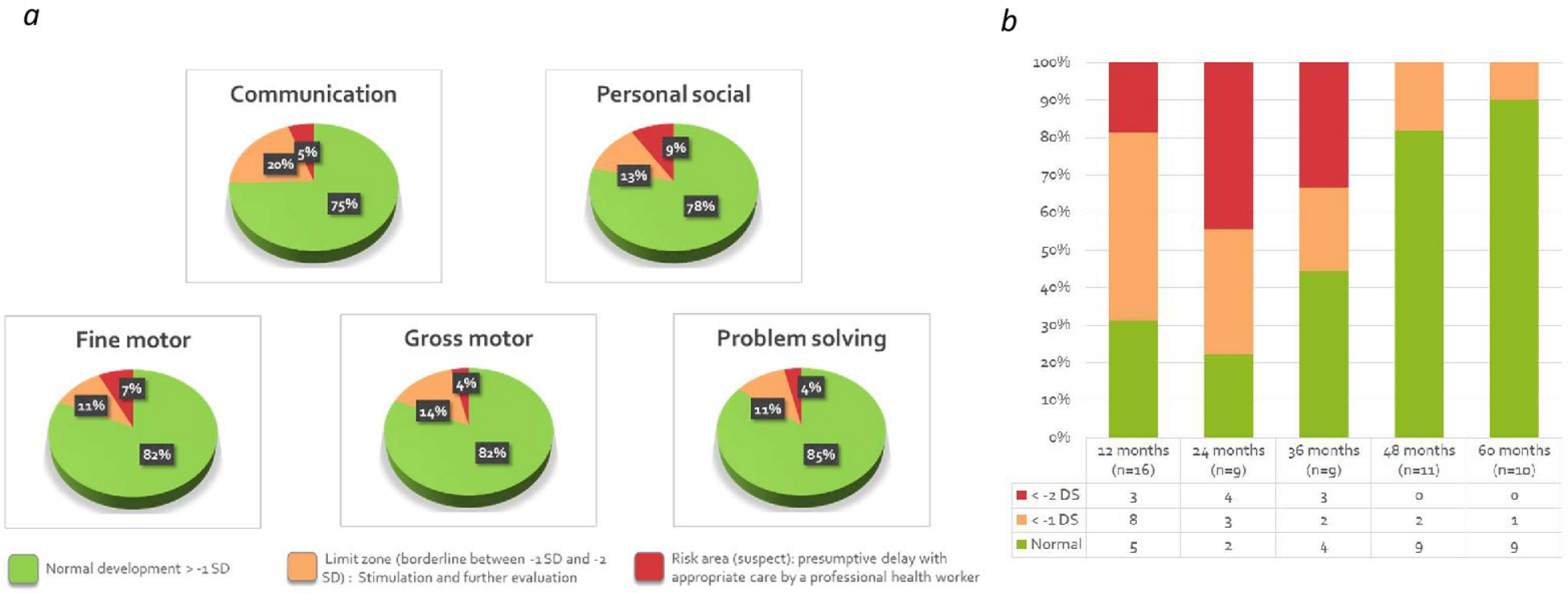
Distribution of the interpretation of numerical scores (normal, borderline, suspect) according to the five different domains (regardless of the realization age) **(a)** and according to the age (regardless of the domains) **(b)**. SD, standard deviation.

The results for the five different axes studied in the questionnaire were also collected separately, as seen in Figure 4a. We observed a lower score in the area of communication and individual and social skills in the 12–36-month age group compared to other areas and at older ages.

Nearly half (47.3%), of these 55 children with a history of PPHN had a test considered as borderline or suspect (prevalence of suspect tests 18%). The ASQ-3 scores of 29 patients (52.7%) were found to be normal, mainly in the age portion between 48 and 60 months (85.7%). Although there is a decrease in the severity of the potential psychomotor delay with age, the percentage of abnormal tests in the 12–24 month cluster was 72% (18 out of 25). Figure 4b shows the distribution of developmental delay based on severity and the age frame of reference.

Within our population, 29 children had benefited from follow-up within the framework of the network RPO. Two of these children stopped the follow-up early after their parents’ refusal, while both having total scores lower than the 10th percentile. Half of the children whose scores were abnormal on the ASQ-3 questionnaire were not initially included in a follow-up programme (13/26).

In our cohort, 27 children (49.09%) were or have been treated in rehabilitation with interactive sessions of physiotherapy, psychomotor or speech therapy, for example. We divided these children into three levels of care: 16 (59.26%) with a single rehabilitation follow-up, 3 (11.1%) worked with two different paramedical professionals and 8 (29.6%) were either integrated in a specialised centre (CAMSP “Centre d'action médico-sociale précoce”, CMP “Centre médicopsychologique” or specialized center for handicap management) or follow at least three rehabilitative treatments. It should also be noted that nearly half (44.4%) of these children received speech therapy. Among the children with a borderline or suspect ASQ-3 questionnaire score in one or more areas, 34.6% (9/26) have never had any rehabilitation treatment and have not been evaluated by a health professional with the aim of a screening from psychomotor development delay.

Out of the 29 normal ASQs, 10 children underwent rehabilitation, two of which were at the highest level of care. About a quarter (25.93%) of the children who required rehabilitation were not included in a follow-up program on discharge from hospitalization.

Lastly, three quarters (39) of the children developed their social skills within a community of children at the time of the questionnaire's realization.

#### Secondary objective

Univariate analysis of ASQ-3 results according to normal or abnormal status (borderline or suspect) for all domains and for each domain is presented in Table 2. We observed a significant difference between the minimum preductal saturation for the overall score (82.2% ± −9.8 in the group overall normal score vs. 75.4% ± 13.4 in the abnormal one, *p* = 002). It was also significant for the areas of communication and fine motor skills. We highlighted a correlation of Pearson between the minimal preductal saturation and the overall ASQ-3 score (positive correlation with a coefficient of 0.41, *p* = 0.002), the communication score (coefficient 0.48, *p* = 0.0002) and the individual and social skills (coefficient 0.38, *p* = 0.0046). The lowest the preductal saturation was, the lower these different scores were. Regarding the fine motor score, it would seem that it was significantly related to the duration of resuscitation (−0.32 with *p* = 0.0166), length of hospitalization (−0.35 with *p* = 0.0096), mechanical ventilation (−0.30 with *p* = 0.0273) and sedation (−0.31 with *p* = 0.0243): the more these periods of time increase, the lower the fine motor score was.

**Table 2:** Univariate analysis of ASQ-3 results according to normal or abnormal status (borderline or suspect) for all domains (A) and for each domain (B) according to the different retrospective quantitative variables.

(A)			
Results of ASQ-3	Normal	Borderline or Suspect	*P*-value
Maternal and neonatal data			
Maternal age (years)	31.4 ± 5.5	32.8 ± 5.1	0.35
Gestational age (WGA^a^)	38.8 ± 2.4	39.4 ± 1.9	0.39
Birth weight (kg)	3.0 ± 0.4	3.3 ± 0.6	**0**.**03**
Head circumference at birth (cm)	34.4 ± 1.6	35.4 ± 1.6	**0**.**03**
Apgar score at 1 min	5.9 ± 3.6	5.9 ± 4.0	0.84
Cord arterial pH	7.2 ± 0.1	7.1 ± 0.2	**0**.**02**
Cord lactate (mmol/L)	6.3 ± 4.0	7.1 ± 3.8	0.42
Clinical and paraclinical data during ICU			
Minimal pre-ductal saturation (%)	82.2 ± 9.8	75.4 ± 13.4	**0**.**02**
Cumulative desaturation score	7.2 ± 10.3	9.1 ± 11.4	0.41
Minimal MBP^b^ (mmHg)	31.3 ± 6.3	30.4 ± 5.4	0.47
Cumulative hypotension score	3.1 ± 4.5	3.9 ± 3.7	0.14
Management and evolution data			
Duration of intensive care stay (days)	11.2 ± 9.1	12.2 ± 15.6	0.42
Total length of hospital stay (days)	24.5 ± 16.8	24.9 ± 24.5	0.51
VIS^c^	59.1 ± 83.7	78.3 ± 98.5	0.41
Dobutamine dosage (*N* = 22) (µg/kg/min)	8.7 ± 2.0	12.1 ± 4.5	0.06
Duration of Dobutamine (days)	1.1 ± 1.6	1.9 ± 2.6	0.46
Total duration of iNO^d^ (days)	83.2 ± 59.4	88.0 ± 90.8	0.69
Total duration of mechanical ventilation (days)	169.7 ± 129.0	204.4 ± 248.5	0.80
Total duration of sedation (days)	5.3 ± 3.8	6.9 ± 7.2	0.32

Bold values indicate significant *p*-values < 0.05.

**Table:** 

(B)												
Results of ASQ-3	Communication			Gross motor			Fine motor			Problem solving		
	Normal	Borderline or suspect	*P*-value	Normal	Borderline or suspect	*P*-value	Normal	Borderline or suspect	*P*-value	Normal	Borderline or suspect	*P*-value
Clinical and paraclinical data												
Minimal pre-ductal saturation (%)	82.1 ± 9.5	69.9 ± 14.2	**0**.**001**	79.9 ± 11.3	74.7 ± 14.7	0.23	80.9 ± 10.5	70.2 ± 14.7	**0**.**008**	78.9 ± 11.9	79.4 ± 13.4	0.90
Management and evolution data												
Duration of intensive care stay (days)	11.2 ± 11.0	13.2 ± 16.4	0.54	11.1 ± 10.6	14.5 ± 19.3	0.73	9.8 ± 7.7	20.2 ± 23.5	0.14	11.0 ± 11.5	15.6 ± 17.9	0.38
Total length of hospital stay (days)	24.2 ± 18.5	26.2 ± 26.4	0.71	39.1 ± 64.8	97.2 ± 132.1	0.29	21.7 ± 15.2	38.1 ± 34.1	0.13	23.4 ± 19.2	32.3 ± 27.9	0.57
VIS^c^	35.5 ± 66.0	91.0 ± 112.1	0.08	51.7 ± 70.2	161.9 ± 137.1	0.09	58.3 ± 79.2	115.2 ± 128.6	0.22	59.2 ± 86.7	110.9 ± 102.1	0.15
Dobutamine dosage (*N* = 22) (µg/kg/min)	9.4 ± 3.6	12.0 ± 3.9	0.09	9.3 ± 2.5	15.0 ± 5.8	**0**.**04**	9.8 ± 3.3	12.3 ± 5.2	0.32	9.4 ± 3.4	13.0 ± 4.0	**0**.**02**
Duration of Dobutamine (days)	1.0 ± 1.6	2.8 ± 3.0	**0**.**03**	1.3 ± 1.8	2.2 ± 3.3	0.65	1.2 ± 1.7	2.8 ± 3.4	0.17	1.2 ± 2.0	3.1 ± 2.5	**0**.**015**
Total duration of iNO^d^ (days)	74.1 ± 54.9	118.9 ± 112.3	0.19	12.6 ± 5.4	11.8 ± 2.8	0.92	76.2 ± 56.5	127.5 ± 126.2	0.30	84.2 ± 75.6	93.0 ± 77.8	0.95
Total duration of mechanical ventilation (days)	163.6 ± 136.1	237.4 ± 303.9	0.28	170.1 ± 129.3	254.6 ± 363.2	0.81	155.7 ± 110.4	318.0 ± 369.1	0.06	180.0 ± 196.9	218.9 ± 173.2	0.29
Total duration of sedation (days)	5.4 ± 4.5	7.6 ± 8.1	0.32	5.7 ± 4.2	8.0 ± 9.8	0.91	5.0 ± 3.4	10.8 ± 9.9	**0**.**02**	5.8 ± 5.5	7.6 ± 6.4	0.35

NB, for quantitative variables with a normal distribution, student test otherwise Wilcoxon (Mann-Whitney). *p* is significant if *p* < 0.05.

^a^ WGA, weeks of gestational age.

^b^ MBP, mean blood pressure.

^c^ VIS, vasoactive-inotropic score.

^d^ iNO, inhaled nitric oxide.

Bold values indicate significant *p*-values < 0.05.

Althought the hemodynamic evolution did not seem to influence the neurological evolution of these children (the VIS score, the minimum MBP, or the cumulative hypotension score weren't associated with ASQ-3 global score level), the duration or dosage of Dobutamine appears to be related to pathological outcomes in the areas of communication, gross motor skills, and problem solving.

By means of a linear univariate regression test, we assessed the risk of having an anomaly in the ASQ-3 scores in the event of a low “minimum preductal saturation” with an Odds Ratio of 1.1 with a confidence interval of 95% [1.04; 1.18] (*p* = 0.002) for the overall score, 1.53 [1.22; 1.91] (*p* = 0.0003) for communication, 1.44 [1.12; 1.85] (*p* = 0.004) for fine motor skills, and 1.25 [1.001; 1.56] (*p* = 0.048) for individual social skills. Social conditions of parents were significantly associated with fine motor function [OR: 0.1 (0.012; 0.76) *p* = 0.027]. In the model of multivariate linear regression, minimal preductal saturation was independently associated with low communication score [OR 1.73 (1.14; 2.63) *p* = 0.013] but not with overall score nor others individual scores (Supplementary Table S1).

The limited number of patients treated with ECMO did not allow a statistical analysis of the impact on ASQ-3 results, but the overall scores of the two patients were among the 10% lowest scores (145 for V-A and 170 for V-V) in our study population. We found similar results in these two 36-month-children for each area, particularly in fine motor skills and communication (lower than the 20th percentile). These children received early follow-up and re-education.

We also observed the tendency for a lower ASQ-3 score (overall, communication, gross and fine motor skills, problem solving) for children classified with the following criteria: supra-systemic PPHN (only for the overall score, communication and fine motor skills), presence of a right-left shunt at the level of the FO (for all scores) and of the CA (except for communication and individual and social skills).

We compared the responder and the non-responder populations. The Supplementary Table S2 shows a significant difference regarding the duration of resuscitation, the ventilator characteristics and hemodynamic management, all of which lower for non-responder children, who appear to be less severe.

## Discussion

In our single-center French population of 55 children, we highlighted, in 47% of cases, the presence of at least one anomaly regarding one of the five domains of the ASQ-3 with 29% classified as borderline and 18% as suspect. Although the median of all these global scores being at 250 [220; 285] was high enough to be considered within normal range of an overall neurological development, the analysis of the results by domain showed not inconsiderable disparities for nearly half of these children. Among the five neuropsychomotor development axes, the areas of “*communication*” and “*social and individual skills*” were the most affected with respectively 25.45% and 21.81% of scores below—1 SD.

Our results confirm a significant prevalence of neuro-psychomotor developmental disorders in children with a history of PPHN, higher than the 10%–30% found in the literature's data, although the comparison with the results of previous studies is not easy, in particular with those dating back to the pre-iNO period. In fact, among the randomized control trials by treatment group (iNO vs. iNO ± ECMO vs. placebo), in 2002, Lipkin et al. (16) reported 46% of the overall abnormalities based on a neurological examination, the Bayley Scales Psychomotor and Mental Development (B-PDI and B-MDI) and hearing status in 144 one-year-old children. Rosenberg et al. in 2010 (19), concluded the average behavior and intelligence scores achieved in 109 seven-year-old children were within normal standards, despite a proportion of 16.6% of intellectual quotients (Full Scale Intelligence Quotient, FSIQ) lower than the usual.

Over the last few years, we witnessed an evolution in resuscitation techniques and management of PPHN, the assessment of neuropsychomotor development has broadened to the notion of so-called minor disorders which consider, more individually, communication and language, memory, social and behavioural skills, beyond the notion of physical or mental disability purely based on the global performance score lower than an overall average or a defective physical examination.

Later, Berti et al. (17) argued that 22% and 26% of their cohort of 27 children with PPHN treated with iNO aged of 41 months (12–70) had language (receptive or expressive) and behavioural disorders, without having a pathological Bayley or WPPSI score, which is close to the results in the areas of communication and individual and social skills found in our study.

We chose to use the ASQ-3 to clearly establish a standardized numerical score and allow us to separately quantify and detect a potential delay in each area of development. Despite the drawbacks associated with collecting data via questionnaires with information and selection biases, this parental questionnaire proves easy to use, with a good reproducibility and validity in the North American population, and is considered to be a recommended and reliable screening tool for developmental disorders. Although the ASQ-3 score is not specifically validated for a cohort of PPHN children, in French population, it is nevertheless used in other studies involving comparable vulnerable paediatric populations of term or near term (38–40) neonates such as congenital diaphragmatic hernias (42), congenital heart defects (43, 44), post-ECMO (45) and hypoxic-ischaemic encephalopathy (41), and has demonstrated feasibility in certain populations with a similar socioeconomic status *(Netherlands, Korea, Norway)*.

Roofthooft et al. (46) also used a similar questionnaire, the Child Behaviour CheckList (CBCL) to study behavioural disorders in 55 PPHN surviving patients aged between 1.5–5 and 5–18 years and found, respectively, 14.3% at 19.1% of behavioural disorders.

Despite the limitations associated with retrospective data collection, the perinatal data's description of this population made it possible to ensure a good selection of our subjects, representative of a population suffering from PPHN clinically and echocardiographically confirmed with an index of oxygenation of moderate severity on average. However, we had difficulty extracting hypoxic ischemic encephalopathies, which are often involved in other diagnoses (as meconium aspiration syndrome, but not systematically), and we are aware that there may be an impact of the initial pathology on the score, that we were not able to explore, given the small number of topics and overlapping diagnoses. In addition to that, we obtained a satisfactory response rate to the questionnaire of 81%, but the comparative analysis with the non-responders shows a difference regarding a longer hospital stay, a more active ventilatory and hemodynamic management and a lower mean preductal minimum saturation in responders. This gap could suggest a higher level of severity in responders and it might be possible that the prevalence of these disorders is overestimated. This difference and the average response age, which is slightly higher than the age of the corresponding questionnaire, could counterbalance one another and therefore underestimate the prevalence of disorders.

A major concern is that the results were derived from different patients at different times: it's clearly not a prospective evaluation of the same patients at different ages and we are mindful of the risk of bias linked with this design. Moreover, we found an interesting distribution of disorders depending on the age, not many studies took an interest in long-term neurological outcomes in children with a history of PPHN up to preschool or even school age. As a matter of fact, even though nothing can be concluded in the absence of prospective follow-up, there is a decrease in the prevalence of abnormal scores in the older category with 85% of normal tests in 48–60 months. We can hypothesize about a “simple” delay in learning, normalizing with time and a social setting, but some authors (47, 48) suggest a lack of sensitivity in older age categories while others say possible cultural disparities negatively influencing the results of younger age groups. Troude's French feasibility study (49) regarding the “gross motor skills” domain, at 12 months. Further analysis would be necessary to compare the severity of these two groups or to propose a secondary assessment of the development at five years old for the 12–36-month subgroup.

With regards to the children's follow-up, it was not systematically set up following a hospitalization for PPHN and 51% of the children in this cohort have never been followed by a health professional for the purpose of screening for psychomotor development disorders. Among the children with an abnormal result classified borderline or suspect, half of them did not benefit from this paediatric follow-up and nearly 35% were not treated in rehabilitation. It is worth noting that, among children with a strictly normal ASQ-3 score in all areas, nearly 35% have benefited from rehabilitation, although we cannot directly study the impact of that rehabilitation on ASQ scores, we can only suppose that early rehabilitation of these children plays a part in stimulating neurodevelopment and potentially improving ASQ outcomes. Finally, three quarters of the children who required or require rehabilitation received a prescription subsequent to a specialised follow-up. This suggests the importance of making a prolonged individual follow-up to all children with PPHN more accessible in order to detect and manage, as early as possible, potential neuro-psychomotor development disorders.

It should also be noted that interpreting the results of the ASQ categorize children into two groups: borderline, who require “stimulation, provision of learning activities, and monitoring,” and suspect, who need further evaluation by a healthcare professional with potential rehabilitation. It is important to keep in mind that this study uses a screening tool and not a diagnosis tool to describe the proportion of neuro-psychomotor abnormalities. It would have been interesting to combine this questionnaire with a specialised evaluation by means of a clinical examination and diagnosis tests (50) as Bayley Scale and to compare the results with those of general population as ELFE cohort (51) but we were limited by the experimental conditions. It is therefore difficult to forecast these exploratory results to a larger population of PPHN. However, it seems essential to continue the research on the neurological outcome of these children with PPHN.

The secondary objective of our article that was to analyze and describe the potential perinatal factors linked with the risk of development of neuro-psychomotor disorders: the main element found relates to the variable “minimal preductal saturation”. In fact, the lower the oxygen saturation of the upper right limb, the more the overall, the communication and the personal-social ASQ-3 scores decrease. PPHN might be the cause of diffuse, multifocal hypoxic brain damage, compromising connections between different cortical areas. Potentially induced by neonatal brain damage, underlying neurological deficiencies can be detected as the development of the child requires some age-dependent cognitive and psychomotor complexity. In order to validate this hypothesis and improve the physiopathological knowledge of the impact of PPHN or neurological development, additional studies with a larger number of subjects and a prolonged follow-up period would be necessary.

## Conclusion

In conclusion, this study about children aged between 1 and 5 years treated with iNO for PPHN showed a significant prevalence of abnormal answers in the ASQ-3 hetero-evaluation mainly focused on communication and individual and social skills, predominantly in the three early years of life. Although it is essential to continue research to understand the neuropathological processes and to document the future of these children, the severity of this pathology and the results seem to require the need to include these newborns with PPHN, as soon as they leave the ICU, in a prolonged follow-up program for an early, individual and multidisciplinary care.

## Data Availability

The raw data supporting the conclusions of this article will be made available by the authors, without undue reservation.
