# Supplementary material for: Neurodevelopmental outcome in children between one and five years after persistent pulmonary hypertension of term and near-term newborns
Source: Front Pediatr. 2024 Oct 23;12:1450916. doi: 10.3389/fped.2024.1450916 (PMC11538055; doi:10.3389/fped.2024.1450916)
Supplement: Supplementary file 1 [file Table1.docx]

| **Variables** | **OR** | **CI 95%** | **p** |
| --- | --- | --- | --- |
| Arterial cord pH | 1,002 | 0,994-1,004 | 0,929 |
| Head circumference | 1,028 | 0,951-1,050 | 0,999 |
| Pre-ductal minimal saturation | 1,605 | 1,109-2,321 | 0,015 |
| Advanced therapies *(including epoprostenol and ECMO)* | 0,994 | 0,984-1,005 | 0,317 |

**S Variables**

Supplementary Table 1 : multivariate regression exploring abnormal score of communication

**Variables**
