# Supplementary material for: Neurodevelopmental outcome in children between one and five years after persistent pulmonary hypertension of term and near-term newborns
Source: Front Pediatr. 2024 Oct 23;12:1450916. doi: 10.3389/fped.2024.1450916 (PMC11538055; doi:10.3389/fped.2024.1450916)
Supplement: Supplementary file 2 [file Table2.docx]

Supplementary table 2: Comparison of perinatal data between responders and non-responders

*^1^ p-value < 0.05 significant*

*^2^ MBP: Mean Blood Pressure*

*^3^ Hypotension or desaturation cumulative scores: recording once per hour of one or more desaturation (≤ 90%) or hypotension (MBP ≤ 35 mmHg)*

*^4^ Total duration of maximal FiO2 corresponding to the number of hours with FiO2 ≥ 90 %*

*VIS: Vaso-inotropic score*
