# Supplementary material for: Neurodevelopmental outcome in children between one and five years after persistent pulmonary hypertension of term and near-term newborns
Source: Front Pediatr. 2024 Oct 23;12:1450916. doi: 10.3389/fped.2024.1450916 (PMC11538055; doi:10.3389/fped.2024.1450916)
Supplement: Supplementary file 3 [file Datasheet1.pdf]

ASQ-3™ Ages & Stages Questionnaires®

12 Month Questionnaire

11 months 0 days through 12 months 30 days

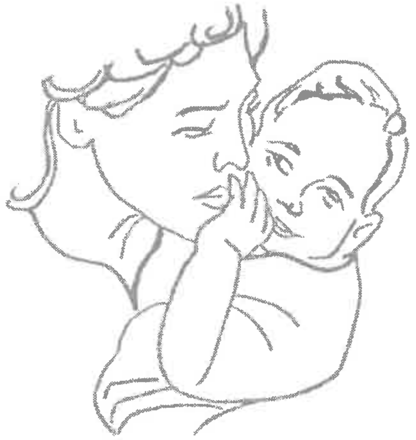

Please provide the following information. Use black or blue ink only and print legibly when completing this form.

Date ASQ completed: ____________________

Baby's information

Baby's first name: ____________________ Middle initial: ______ Baby's last name: ____________________

Baby's date of birth: ____________________

If baby was born 3 or more weeks prematurely, # of weeks premature: ______

Baby's gender: ◯ Male ◯ Female

Person filling out questionnaire

First name: ____________________ Middle initial: ______ Last name: ____________________

Street address: ____________________

Relationship to baby: ◯ Parent ◯ Guardian ◯ Teacher ◯ Child care provider ◯ Grandparent or other relative ◯ Foster parent ◯ Other: ______

City: ____________________ State/ Province: ______ ZIP/ Postal code: ______

Country: ____________________ Home telephone number: ______ Other telephone number: ______

E-mail address: ____________________

Names of people assisting in questionnaire completion: ____________________

Program Information

Baby ID #: ____________________ Age at administration in months and days: ______

Program ID #: ____________________ If premature, adjusted age in months and days: ______

Program name: ____________________

P101120100

© 2009 Paul H. Brookes Publishing Co. All rights reserved.

12 Month Questionnaire

11 months 0 days through 12 months 30 days

On the following pages are questions about activities babies may do. Your baby may have already done some of the activities described here, and there may be some your baby has not begun doing yet. For each item, please fill in the circle that indicates whether your baby is doing the activity regularly, sometimes, or not yet.

Important Points to Remember:

- ☑ Try each activity with your baby before marking a response.
- ☑ Make completing this questionnaire a game that is fun for you and your baby.
- ☑ Make sure your baby is rested and fed.
- ☑ Please return this questionnaire by ______________.

Notes:

____

____

____

____

COMMUNICATION

	YES	SOMETIMES	NOT YET	
1. Does your baby make two similar sounds, such as "ba-ba," "da-da," or "ga-ga"? *(The sounds do not need to mean anything.)*	○	○	○	___
2. If you ask your baby to, does he play at least one nursery game even if you don't show him the activity yourself (such as "bye-bye," "Peeka-boo," "clap your hands," "So Big")?	○	○	○	___
3. Does your baby follow one simple command, such as "Come here," "Give it to me," or "Put it back," *without* your using gestures?	○	○	○	___
4. Does your baby say three words, such as "Mama," "Dada," and "Baba"? *(A "word" is a sound or sounds your baby says consistently to mean someone or something.)*	○	○	○	___
5. When you ask, "Where is the ball (hat, shoe, etc.)?" does your baby look at the object? *(Make sure the object is present. Mark "yes" if she knows one object.)*	○	○	○	___
6. When your baby wants something, does he tell you by *pointing* to it?	○	○	○	___

COMMUNICATION TOTAL ___

GROSS MOTOR

	YES	SOMETIMES	NOT YET	
1. While holding onto furniture, does your baby bend down and pick up a toy from the floor and then return to a standing position? 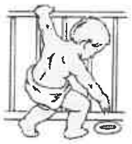	○	○	○	___
2. While holding onto furniture, does your baby lower herself with control (without falling or flopping down)?	○	○	○	___
3. Does your baby walk beside furniture while holding on with only one hand?	○	○	○	___

E101120200

Ages & Stages Questionnaires®, Third Edition (ASQ-3™), Squires & Bricker
© 2009 Paul H. Brookes Publishing Co. All rights reserved.

GROSS MOTOR *(continued)*

		YES	SOMETIMES	NOT YET	
4.	If you hold both hands just to balance your baby, does he take several steps without tripping or falling? *(If your baby already walks alone, mark "yes" for this item.)* 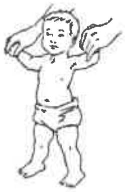	○	○	○	___
5.	When you hold *one hand* just to balance your baby, does she take several steps forward? *(If your baby already walks alone, mark "yes" for this item.)* 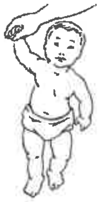	○	○	○	___
6.	Does your baby stand up in the middle of the floor by himself and take several steps forward?	○	○	○	___
			GROSS MOTOR TOTAL		___

FINE MOTOR

		YES	SOMETIMES	NOT YET	
1.	After one or two tries, does your baby pick up a piece of string with his first finger and thumb? *(The string may be attached to a toy.)* 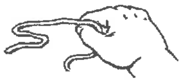	○	○	○	___
2.	Does your baby pick up a crumb or Cheerio with the *tips* of her thumb and a finger? She may rest her arm or hand on the table while doing it. 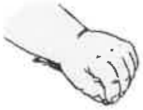	○	○	○	___
3.	Does your baby put a small toy down, without dropping it, and then take his hand off the toy?	○	○	○	___
4.	Without resting her arm or hand on the table, does your baby pick up a crumb or Cheerio with the *tips* of her thumb and a finger? 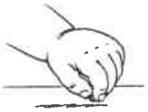	○	○	○	___ *
5.	Does your baby throw a small ball with a forward arm motion? *(If he simply drops the ball, mark "not yet" for this item.)* 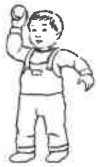	○	○	○	___
6.	Does your baby help turn the pages of a book? *(You may lift a page for him to grasp.)*	○	○	○	___
			FINE MOTOR TOTAL		___

**If Fine Motor Item 4 is marked "yes" or "sometimes," mark Fine Motor Item 2 "yes."*

E101120300

© 2009 Paul H. Brookes Publishing Co. All rights reserved.

PROBLEM SOLVING

	YES	SOMETIMES	NOT YET	
1. When holding a small toy in each hand, does your baby clap the toys together (like "Pat-a-cake")?				
2. Does your baby poke at or try to get a crumb or Cheerio that is inside a clear bottle (such as a plastic soda-pop bottle or baby bottle)?				
3. After watching you hide a small toy under a piece of paper or cloth, does your baby find it? *(Be sure the toy is completely hidden.)*				
4. If you put a small toy into a bowl or box, does your baby copy you by putting in a toy, although she may not let go of it? *(If she already lets go of the toy into a bowl or box, mark "yes" for this item.)*				
5. Does your baby drop two small toys, one after the other, into a container like a bowl or box? *(You may show him how to do it.)*				*
6. After you scribble back and forth on paper with a crayon (or a pencil or pen), does your baby copy you by scribbling? *(If she already scribbles on her own, mark "yes" for this item.)*				

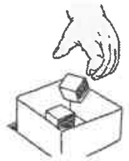

PROBLEM SOLVING TOTAL ____

**If Problem Solving Item 5 is marked "yes" or "sometimes," mark Problem Solving Item 4 "yes."*

PERSONAL-SOCIAL

	YES	SOMETIMES	NOT YET	
1. When you hold out your hand and ask for his toy, does your baby offer it to you even if he doesn't let go of it? *(If he already lets go of the toy into your hand, mark "yes" for this item.)*				
2. When you dress your baby, does she push her arm through a sleeve once her arm is started in the hole of the sleeve?				
3. When you hold out your hand and ask for his toy, does your baby let go of it into your hand?				
4. When you dress your baby, does she lift her foot for her shoe, sock, or pant leg?				
5. Does your baby roll or throw a ball back to you so that you can return it to him?				
6. Does your baby play with a doll or stuffed animal by hugging it?				

PERSONAL-SOCIAL TOTAL ____

E101120400

© 2009 Paul H. Brookes Publishing Co. All rights reserved.

OVERALL

Parents and providers may use the space below for additional comments.

1. Does your baby use both hands and both legs equally well? If no, explain: ○ YES ○ NO

2. Does your baby play with sounds or seem to make words? If no, explain: ○ YES ○ NO

3. When your baby is standing, are her feet flat on the surface most of the time? If no, explain: ○ YES ○ NO

4. Do you have concerns that your baby is too quiet or does not make sounds like other babies do? If yes, explain: ○ YES ○ NO

5. Does either parent have a family history of childhood deafness or hearing impairment? If yes, explain: ○ YES ○ NO

Ages & Stages Questionnaires®, Third Edition (ASQ-3™), Squires & Bricker
© 2009 Paul H. Brookes Publishing Co. All rights reserved.

OVERALL *(continued)*

6. Do you have concerns about your baby's vision? If yes, explain: ◯ YES ◯ NO

7. Has your baby had any medical problems in the last several months? If yes, explain: ◯ YES ◯ NO

8. Do you have any concerns about your baby's behavior? If yes, explain: ◯ YES ◯ NO

9. Does anything about your baby worry you? If yes, explain: ◯ YES ◯ NO

E101120600

Ages & Stages Questionnaires®, Third Edition (ASQ-3™), Squires & Bricker
© 2009 Paul H. Brookes Publishing Co. All rights reserved.

ASQ-3

12 Month ASQ-3 Information Summary

11 months 0 days through 12 months 30 days

Baby's name: ______________________ Date ASQ completed: ______________________

Baby's ID #: ______________________ Date of birth: ______________________

Administering program/provider: ______________________ Was age adjusted for prematurity when selecting questionnaire? ○ Yes ○ No

1. SCORE AND TRANSFER TOTALS TO CHART BELOW: See *ASQ-3 User's Guide* for details, including how to adjust scores if item responses are missing. Score each item (YES = 10, SOMETIMES = 5, NOT YET = 0). Add item scores, and record each area total. In the chart below, transfer the total scores, and fill in the circles corresponding with the total scores.

Area	Cutoff	Total Score	0	5	10	15	20	25	30	35	40	45	50	55	60
Communication	15.64		○	○	○	○	○	○	○	○	○	○	○	○	○
Gross Motor	21.49		○	○	○	○	○	○	○	○	○	○	○	○	○
Fine Motor	34.50		○	○	○	○	○	○	○	○	○	○	○	○	○
Problem Solving	27.32		○	○	○	○	○	○	○	○	○	○	○	○	○
Personal-Social	21.73		○	○	○	○	○	○	○	○	○	○	○	○	○

2. TRANSFER OVERALL RESPONSES: Bolded uppercase responses require follow-up. See *ASQ-3 User's Guide*, Chapter 6.

1. Uses both hands and both legs equally well? Yes **NO**
   Comments:
2. Plays with sounds or seems to make words? Yes **NO**
   Comments:
3. Feet are flat on the surface most of the time? Yes **NO**
   Comments:
4. Concerns about not making sounds? **YES** No
   Comments:
5. Family history of hearing impairment? **YES** No
   Comments:
6. Concerns about vision? **YES** No
   Comments:
7. Any medical problems? **YES** No
   Comments:
8. Concerns about behavior? **YES** No
   Comments:
9. Other concerns? **YES** No
   Comments:

3. ASQ SCORE INTERPRETATION AND RECOMMENDATION FOR FOLLOW-UP: You must consider total area scores, overall responses, and other considerations, such as opportunities to practice skills, to determine appropriate follow-up.

If the baby's total score is in the ☐ (white) area, it is above the cutoff, and the baby's development appears to be on schedule.
If the baby's total score is in the ☐ (gray) area, it is close to the cutoff. Provide learning activities and monitor.
If the baby's total score is in the ■ (black) area, it is below the cutoff. Further assessment with a professional may be needed.

4. FOLLOW-UP ACTION TAKEN: Check all that apply.

_____ Provide activities and rescreen in _____ months.

_____ Share results with primary health care provider.

_____ Refer for (circle all that apply) hearing, vision, and/or behavioral screening.

_____ Refer to primary health care provider or other community agency (specify reason): ______________________.

_____ Refer to early intervention/early childhood special education.

_____ No further action taken at this time

_____ Other (specify): ______________________

5. OPTIONAL: Transfer item responses (Y = YES, S = SOMETIMES, N = NOT YET, X = response missing).

	1	2	3	4	5	6
Communication						
Gross Motor						
Fine Motor						
Problem Solving						
Personal-Social						

P101120700

Ages & Stages Questionnaires®, Third Edition (ASQ-3™), Squires & Bricker
© 2009 Paul H. Brookes Publishing Co. All rights reserved.
