# Supplementary material for: Neurodevelopmental outcome in children between one and five years after persistent pulmonary hypertension of term and near-term newborns
Source: Front Pediatr. 2024 Oct 23;12:1450916. doi: 10.3389/fped.2024.1450916 (PMC11538055; doi:10.3389/fped.2024.1450916)
Supplement: Supplementary file 4 [file Datasheet2.pdf]

ASQ-3™

Ages & Stages Questionnaires®

23 months 0 days through 25 months 15 days

24 Month Questionnaire

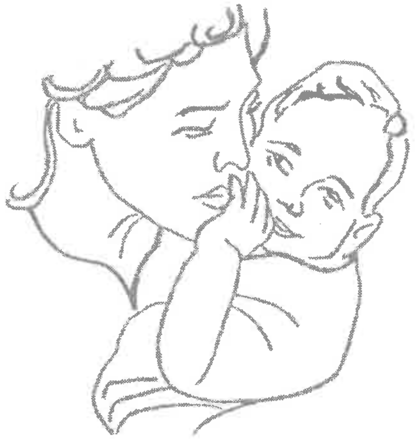

Please provide the following information. Use black or blue ink only and print legibly when completing this form.

Date ASQ completed: ______

Child's information

Child's first name: ______ Middle initial: ______ Child's last name: ______

Child's date of birth: ______

Child's gender: ◯ Male ◯ Female

Person filling out questionnaire

First name: ______ Middle initial: ______ Last name: ______

Street address: ______

Relationship to child:
◯ Parent ◯ Guardian ◯ Teacher ◯ Child care provider
◯ Grandparent or other relative ◯ Foster parent ◯ Other: ______

City: ______ State/Province: ______ ZIP/Postal code: ______

Country: ______ Home telephone number: ______ Other telephone number: ______

E-mail address: ______

Names of people assisting in questionnaire completion: ______

______

Program Information

Child ID #: ______

Program ID #: ______

Program name: ______

P101240100

Ages & Stages Questionnaires®, Third Edition (ASQ-3™), Squires & Bricker
© 2009 Paul H. Brookes Publishing Co. All rights reserved.

ASQ-3™

24 Month Questionnaire

23 months 0 days through 25 months 15 days

On the following pages are questions about activities babies may do. Your baby may have already done some of the activities described here, and there may be some your baby has not begun doing yet. For each item, please fill in the circle that indicates whether your baby is doing the activity regularly, sometimes, or not yet.

Important Points to Remember:

- ☑ Try each activity with your baby before marking a response.
- ☑ Make completing this questionnaire a game that is fun for you and your child.
- ☑ Make sure your child is rested and fed.
- ☑ Please return this questionnaire by ______________.

Notes:

____

____

____

____

At this age, many toddlers may not be cooperative when asked to do things. You may need to try the following activities with your child more than one time. If possible, try the activities when your child is cooperative. If your child can do the activity but refuses, mark "yes" for the item.

COMMUNICATION

	YES	SOMETIMES	NOT YET	
1. Without your showing him, does your child *point* to the correct picture when you say, "Show me the kitty," or ask, "Where is the dog?" *(She needs to identify only one picture correctly.)*	○	○	○	___
2. Does your child imitate a two-word sentence? For example, when you say a two-word phrase, such as "Mama eat," "Daddy play," "Go home," or "What's this?" does your child say both words back to you? *(Mark "yes" even if her words are difficult to understand.)*	○	○	○	___
3. Without your giving him clues by pointing or using gestures, can your child carry out at least *three* of these kinds of directions?	○	○	○	___

- ○ a. "Put the toy on the table."
- ○ b. "Close the door."
- ○ c. "Bring me a towel."
- ○ d. "Find your coat."
- ○ e. "Take my hand."
- ○ f. "Get your book."

	YES	SOMETIMES	NOT YET	
4. If you point to a picture of a ball (kitty, cup, hat, etc.) and ask your child, "What is this?" does your child correctly *name* at least one picture?	○	○	○	___
5. Does your child say two or three words that represent different ideas together, such as "See dog," "Mommy come home," or "Kitty gone"? *(Don't count word combinations that express one idea, such as "bye-bye," "all gone," "all right," and "What's that?")* Please give an example of your child's word combinations:	○	○	○	___

E101240200

Ages & Stages Questionnaires®, Third Edition (ASQ-3™), Squires & Bricker
© 2009 Paul H. Brookes Publishing Co. All rights reserved.

COMMUNICATION *(continued)*

	YES	SOMETIMES	NOT YET	
6. Does your child correctly use at least two words like "me," "I," "mine," and "you"?				
		COMMUNICATION TOTAL		

GROSS MOTOR

	YES	SOMETIMES	NOT YET	
1. Does your child walk down stairs if you hold onto one of her hands? She may also hold onto the railing or wall. *(You can look for this at a store, on a playground, or at home.)*				
2. When you show your child how to kick a large ball, does he try to kick the ball by moving his leg forward or by walking into it? *(If your child already kicks a ball, mark "yes" for this item.)* 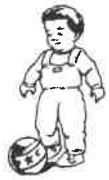				
3. Does your child walk either up or down at least two steps by herself? She may hold onto the railing or wall. 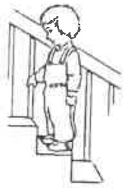				
4. Does your child run fairly well, stopping herself without bumping into things or falling? 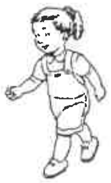				
5. Does your child jump with both feet leaving the floor at the same time? 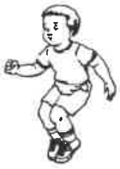				
6. Without holding onto anything for support, does your child kick a ball by swinging his leg forward? 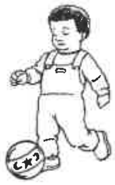				*
		GROSS MOTOR TOTAL		

**If Gross Motor Item 6 is marked "yes" or "sometimes," mark Gross Motor Item 2 "yes."*

E101240300

Ages & Stages Questionnaires®, Third Edition (ASQ-3™), Squires & Bricker
© 2009 Paul H. Brookes Publishing Co. All rights reserved.

FINE MOTOR

	YES	SOMETIMES	NOT YET	
1. Does your child get a spoon into his mouth right side up so that the food usually doesn't spill?	○	○	○	___
2. Does your child turn the pages of a book by herself? *(She may turn more than one page at a time.)*	○	○	○	___
3. Does your child use a turning motion with his hand while trying to turn doorknobs, wind up toys, twist tops, or screw lids on and off jars?	○	○	○	___
4. Does your child flip switches off and on?	○	○	○	___
5. Does your child stack seven small blocks or toys on top of each other by herself? *(You could also use spools of thread, small boxes, or toys that are about 1 inch in size.)*	○	○	○	___
6. Can your child string small items such as beads, macaroni, or pasta "wagon wheels" onto a string or shoelace?	○	○	○	___

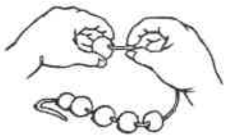

FINE MOTOR TOTAL ___

PROBLEM SOLVING

Count as "yes"

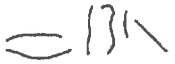

Count as "not yet"

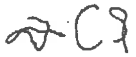

	YES	SOMETIMES	NOT YET	
1. After watching you draw a line from the top of the paper to the bottom with a crayon (or pencil or pen), does your child copy you by drawing a single line on the paper in *any direction*? *(Mark "not yet" if your child scribbles back and forth.)*	○	○	○	___
2. After a crumb or Cheerio is dropped into a small, clear bottle, does your child turn the bottle upside down to dump out the crumb or Cheerio? *(Do not show him how.) (You can use a soda-pop bottle or baby bottle.)*	○	○	○	___
3. Does your child pretend objects are something else? For example, does your child hold a cup to her ear, pretending it is a telephone? Does she put a box on her head, pretending it is a hat? Does she use a block or small toy to stir food?	○	○	○	___
4. Does your child put things away where they belong? For example, does he know his toys belong on the toy shelf, his blanket goes on his bed, and dishes go in the kitchen?	○	○	○	___
5. If your child wants something she cannot reach, does she find a chair or box to stand on to reach it (for example, to get a toy on a counter or to "help" you in the kitchen)?	○	○	○	___

E101240400

© 2009 Paul H. Brookes Publishing Co. All rights reserved.

PROBLEM SOLVING *(continued)*

	YES	SOMETIMES	NOT YET	
6. While your child watches, line up four objects like blocks or cars in a row. Does your child copy or imitate you and line up *four* objects in a row? *(You can also use spools of thread, small boxes, or other toys.)* 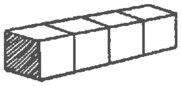	○	○	○	___

PROBLEM SOLVING TOTAL ___

PERSONAL-SOCIAL

	YES	SOMETIMES	NOT YET	
1. Does your child drink from a cup or glass, putting it down again with little spilling?	○	○	○	___
2. Does your child copy the activities you do, such as wipe up a spill, sweep, shave, or comb hair?	○	○	○	___
3. Does your child eat with a fork?	○	○	○	___
4. When playing with either a stuffed animal or a doll, does your child pretend to rock it, feed it, change its diapers, put it to bed, and so forth?	○	○	○	___
5. Does your child push a little wagon, stroller, or other toy on wheels, steering it around objects and backing out of corners if he cannot turn?	○	○	○	___
6. Does your child call herself "I" or "me" more often than her own name? For example, "I do it," more often than "Juanita do it."	○	○	○	___

PERSONAL-SOCIAL TOTAL ___

OVERALL

Parents and providers may use the space below for additional comments.

1. Do you think your child hears well? If no, explain: ○ YES ○ NO

2. Do you think your child talks like other toddlers her age? If no, explain: ○ YES ○ NO

E101240500

Ages & Stages Questionnaires®, Third Edition (ASQ-3™), Squires & Bricker
© 2009 Paul H. Brookes Publishing Co. All rights reserved.

OVERALL *(continued)*

3. Can you understand most of what your child says? If no, explain: ◯ YES ◯ NO

4. Do you think your child walks, runs, and climbs like other toddlers his age? If no, explain: ◯ YES ◯ NO

5. Does either parent have a family history of childhood deafness or hearing impairment? If yes, explain: ◯ YES ◯ NO

6. Do you have any concerns about your child's vision? If yes, explain: ◯ YES ◯ NO

7. Has your child had any medical problems in the last several months? If yes, explain: ◯ YES ◯ NO

Ages & Stages Questionnaires®, Third Edition (ASQ-3™), Squires & Bricker
© 2009 Paul H. Brookes Publishing Co. All rights reserved.

OVERALL *(continued)*

8. Do you have any concerns about your child's behavior? If yes, explain: ◯ YES ◯ NO

9. Does anything about your child worry you? If yes, explain: ◯ YES ◯ NO

Ages & Stages Questionnaires®, Third Edition (ASQ-3™), Squires & Bricker
© 2009 Paul H. Brookes Publishing Co. All rights reserved.

ASQ-3 24 Month ASQ-3 Information Summary

23 months 0 days through 25 months 15 days

Child's name: ______________________ Date ASQ completed: ______________________

Child's ID #: ______________________ Date of birth: ______________________

Administering program/provider: ______________________

1. **SCORE AND TRANSFER TOTALS TO CHART BELOW:** See *ASQ-3 User's Guide* for details, including how to adjust scores if item responses are missing. Score each item (YES = 10, SOMETIMES = 5, NOT YET = 0). Add item scores, and record each area total. In the chart below, transfer the total scores, and fill in the circles corresponding with the total scores.

Area	Cutoff	Total Score	0	5	10	15	20	25	30	35	40	45	50	55	60
Communication	25.17														
Gross Motor	38.07														
Fine Motor	35.16														
Problem Solving	29.78														
Personal-Social	31.54														

2. **TRANSFER OVERALL RESPONSES:** Bolded uppercase responses require follow-up. See *ASQ-3 User's Guide*, Chapter 6.

1. Hears well? Yes **NO**
   Comments:
2. Talks like other toddlers his age? Yes **NO**
   Comments:
3. Understand most of what your child says? Yes **NO**
   Comments:
4. Walks, runs, and climbs like other toddlers? Yes **NO**
   Comments:
5. Family history of hearing impairment? **YES** No
   Comments:
6. Concerns about vision? **YES** No
   Comments:
7. Any medical problems? **YES** No
   Comments:
8. Concerns about behavior? **YES** No
   Comments:
9. Other concerns? **YES** No
   Comments:

3. **ASQ SCORE INTERPRETATION AND RECOMMENDATION FOR FOLLOW-UP:** You must consider total area scores, overall responses, and other considerations, such as opportunities to practice skills, to determine appropriate follow-up.

If the child's total score is in the ☐ area, it is above the cutoff, and the child's development appears to be on schedule.
If the child's total score is in the ▒ area, it is close to the cutoff. Provide learning activities and monitor.
If the child's total score is in the ■ area, it is below the cutoff. Further assessment with a professional may be needed.

4. **FOLLOW-UP ACTION TAKEN:** Check all that apply.

______ Provide activities and rescreen in _____ months.
______ Share results with primary health care provider.
______ Refer for (circle all that apply) hearing, vision, and/or behavioral screening.
______ Refer to primary health care provider or other community agency (specify reason): ______________________.
______ Refer to early intervention/early childhood special education.
______ No further action taken at this time
______ Other (specify): ______________________

5. **OPTIONAL:** Transfer item responses (Y = YES, S = SOMETIMES, N = NOT YET, X = response missing).

	1	2	3	4	5	6
Communication						
Gross Motor						
Fine Motor						
Problem Solving						
Personal-Social						

P101240800

© 2009 Paul H. Brookes Publishing Co. All rights reserved.
