# Supplementary material for: Neurodevelopmental outcome in children between one and five years after persistent pulmonary hypertension of term and near-term newborns
Source: Front Pediatr. 2024 Oct 23;12:1450916. doi: 10.3389/fped.2024.1450916 (PMC11538055; doi:10.3389/fped.2024.1450916)
Supplement: Supplementary file 5 [file Datasheet3.pdf]

ASQ-3™

Ages & Stages Questionnaires®

45 months 0 days through 50 months 30 days

48 Month Questionnaire

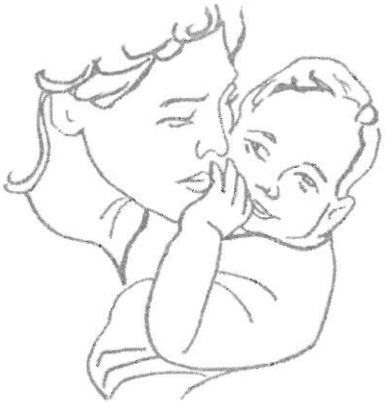

Please provide the following information. Use black or blue ink only and print legibly when completing this form.

Date ASQ completed: M M D D Y Y Y Y

Child's information

Child's first name:

Middle initial:

Child's last name:

Child's date of birth: M M D D Y Y Y Y

Child's gender:
- ◯ Male
- ◯ Female

Person filling out questionnaire

First name:

Middle initial:

Last name:

Street address:

Relationship to child:
- ◯ Parent
- ◯ Guardian
- ◯ Teacher
- ◯ Child care provider
- ◯ Grandparent or other relative
- ◯ Foster parent
- ◯ Other:

City:

State/Province:

ZIP/Postal code:

Country:

Home telephone number:

Other telephone number:

E-mail address:

Names of people assisting in questionnaire completion:

PROGRAM INFORMATION

Child ID #:

Program ID #:

Program name:

E101480100

Ages & Stages Questionnaires®, Third Edition (ASQ-3™), Squires & Bricker
© 2009 Paul H. Brookes Publishing Co. All rights reserved.

ASQ-3™

48 Month Questionnaire

45 months 0 days through 50 months 30 days

On the following pages are questions about activities children may do. Your child may have already done some of the activities described here, and there may be some your child has not begun doing yet. For each item, please fill in the circle that indicates whether your child is doing the activity regularly, sometimes, or not yet.

Important Points to Remember:

- ☑ Try each activity with your child before marking a response.
- ☑ Make completing this questionnaire a game that is fun for you and your child.
- ☑ Make sure your child is rested and fed.
- ☑ Please return this questionnaire by ____________.

Notes:

____

____

____

____

COMMUNICATION

	YES	SOMETIMES	NOT YET	
1. Does your child name at least three items from a common category? For example, if you say to your child, "Tell me some things that you can eat," does your child answer with something like "cookies, eggs, and cereal"? Or if you say, "Tell me the names of some animals," does your child answer with something like "cow, dog, and elephant"?	○	○	○	___
2. Does your child answer the following questions? *(Mark "sometimes" if your child answers only one question.)*	○	○	○	___

"What do you do when you are hungry?" *(Acceptable answers include "get food," "eat," "ask for something to eat," and "have a snack.")* Please write your child's response:

"What do you do when you are tired?" *(Acceptable answers include "take a nap," "rest," "go to sleep," "go to bed," "lie down," and "sit down.")* Please write your child's response:

	YES	SOMETIMES	NOT YET	
3. Does your child tell you at least two things about common objects? For example, if you say to your child, "Tell me about your ball," does she say something like, "It's round. I throw it. It's big"?	○	○	○	___
4. Does your child use endings of words, such as "-s," "-ed," and "-ing"? For example, does your child say things like, "I see two cats," "I am playing," or "I kicked the ball"?	○	○	○	___

Ages & Stages Questionnaires®, Third Edition (ASQ-3™), Squires & Bricker
© 2009 Paul H. Brookes Publishing Co. All rights reserved.

COMMUNICATION *(continued)*

	YES	SOMETIMES	NOT YET	
5. Without your giving help by pointing or repeating, does your child follow three directions that are *unrelated* to one another? Give all three directions before your child starts. For example, you may ask your child, "Clap your hands, walk to the door, and sit down," or "Give me the pen, open the book, and stand up."	○	○	○	___
6. Does your child use all of the words in a sentence (for example, "a," "the," "am," "is," and "are") to make complete sentences, such as "I *am* going to *the* park," or "*Is* there *a* toy to play with?" or "*Are* you coming, too?"	○	○	○	___
		COMMUNICATION TOTAL		___

GROSS MOTOR

	YES	SOMETIMES	NOT YET	
1. Does your child catch a large ball with both hands? *(You should stand about 5 feet away and give your child two or three tries before you mark the answer.)* 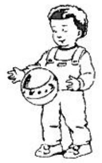	○	○	○	___
2. Does your child climb the rungs of a ladder of a playground slide and slide down without help?	○	○	○	___
3. While standing, does your child throw a ball *overhand* in the direction of a person standing at least 6 feet away? To throw overhand, your child must raise his arm to shoulder height and throw the ball forward. *(Dropping the ball or throwing the ball underhand should be scored as "not yet.")* 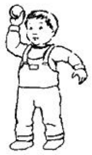	○	○	○	___
4. Does your child hop up and down on either the right or left foot at least one time without losing her balance or falling?	○	○	○	___
5. Does your child jump forward a distance of 20 inches from a standing position, starting with his feet together?	○	○	○	___
6. Without holding onto anything, does your child stand on one foot for at least 5 seconds without losing her balance and putting her foot down? *(You may give your child two or three tries before you mark the answer.)* 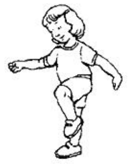	○	○	○	___
		GROSS MOTOR TOTAL		___

FINE MOTOR

	YES	SOMETIMES	NOT YET	
1. Does your child put together a five- to seven-piece interlocking puzzle? *(If one is not available, take a full-page picture from a magazine or catalog and cut it into six pieces. Does your child put it back together correctly?)*	○	○	○	___

Ages & Stages Questionnaires®, Third Edition (ASQ-3™), Squires & Bricker
© 2009 Paul H. Brookes Publishing Co. All rights reserved.

FINE MOTOR *(continued)*

	YES	SOMETIMES	NOT YET	
2. Using child-safe scissors, does your child cut a paper in half on a more or less straight line, making the blades go up and down? *(Carefully watch your child's use of scissors for safety reasons.)* 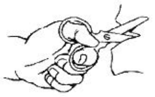	○	○	○	___
3. Using the shapes below to look at, does your child copy at least three shapes onto a large piece of paper using a pencil, crayon, or pen, without tracing? *(Your child's drawings should look similar to the design of the shapes below, but they may be different in size.)* 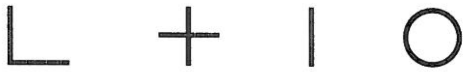	○	○	○	___
4. Does your child unbutton one or more buttons? *(Your child may use his own clothing or a doll's clothing.)*	○	○	○	___
5. Does your child draw pictures of people that have at least three of the following features: head, eyes, nose, mouth, neck, hair, trunk, arms, hands, legs, or feet?	○	○	○	___
6. Does your child color mostly within the lines in a coloring book or within the lines of a 2-inch circle that you draw? *(Your child should not go more than 1/4 inch outside the lines on most of the picture.)*	○	○	○	___
		FINE MOTOR TOTAL		___

PROBLEM SOLVING

	YES	SOMETIMES	NOT YET	
1. When you say, "Say 'five eight three,'" does your child repeat *just* the three numbers in the same order? *Do not repeat the numbers.* If necessary, try another series of numbers and say, "Say 'six nine two.'" *(Your child must repeat just one series of three numbers to answer "yes" to this question.)*	○	○	○	___
2. When asked, "Which circle is the smallest?" does your child point to the smallest circle? *(Ask this question without providing help by pointing, gesturing, or looking at the smallest circle.)* 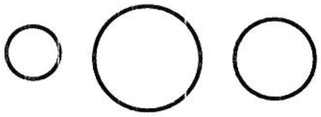	○	○	○	___
3. Without your giving help by pointing, does your child follow three different directions using the words "under," "between," and "middle"? For example, ask your child to put the shoe "*under* the couch." Then ask her to put the ball "*between* the chairs" and the book "in the *middle* of the table."	○	○	○	___
4. When shown objects and asked, "What color is this?" does your child name five different colors, like red, blue, yellow, orange, black, white, or pink? *(Mark "yes" only if your child answers the question correctly using five colors.)*	○	○	○	___

Ages & Stages Questionnaires®, Third Edition (ASQ-3™), Squires & Bricker
© 2009 Paul H. Brookes Publishing Co. All rights reserved.

PROBLEM SOLVING *(continued)*

	YES	SOMETIMES	NOT YET	
5. Does your child dress up and "play-act," pretending to be someone or something else? For example, your child may dress up in different clothes and pretend to be a mommy, daddy, brother, or sister, or an imaginary animal or figure.	○	○	○	___
6. If you place five objects in front of your child, can he count them by saying, "one, two, three, four, five," in order? *(Ask this question without providing help by pointing, gesturing, or naming.)*	○	○	○	___
		PROBLEM SOLVING TOTAL		___

PERSONAL-SOCIAL

	YES	SOMETIMES	NOT YET	
1. Does your child serve herself, taking food from one container to another using utensils? For example, does your child use a large spoon to scoop applesauce from a jar into a bowl?	○	○	○	___
2. Does your child tell you at least four of the following? Please mark the items your child knows.	○	○	○	___

○ a. First name ○ d. Last name

○ b. Age ○ e. Boy or girl

○ c. City she lives in ○ f. Telephone number

	YES	SOMETIMES	NOT YET	
3. Does your child wash his hands using soap and water and dry off with a towel without help?	○	○	○	___
4. Does your child tell you the names of two or more playmates, not including brothers and sisters? *(Ask this question without providing help by suggesting names of playmates or friends.)*	○	○	○	___
5. Does your child brush her teeth by putting toothpaste on the toothbrush and brushing all of her teeth without help? *(You may still need to check and rebrush your child's teeth.)*	○	○	○	___
6. Does your child dress or undress himself without help (except for snaps, buttons, and zippers)?	○	○	○	___
		PERSONAL-SOCIAL TOTAL		___

OVERALL

Parents and providers may use the space below for additional comments.

1. Do you think your child hears well? If no, explain: ○ YES ○ NO

E101480500

Ages & Stages Questionnaires®, Third Edition (ASQ-3™), Squires & Bricker
© 2009 Paul H. Brookes Publishing Co. All rights reserved.

OVERALL *(continued)*

2. Do you think your child talks like other children her age? If no, explain: ○ YES ○ NO

3. Can you understand most of what your child says? If no, explain: ○ YES ○ NO

4. Can other people understand most of what your child says? If no, explain: ○ YES ○ NO

5. Do you think your child walks, runs, and climbs like other children his age? If no, explain: ○ YES ○ NO

6. Does either parent have a family history of childhood deafness or hearing impairment? If yes, explain: ○ YES ○ NO

7. Do you have any concerns about your child's vision? If yes, explain: ○ YES ○ NO

Ages & Stages Questionnaires®, Third Edition (ASQ-3™), Squires & Bricker
© 2009 Paul H. Brookes Publishing Co. All rights reserved.

OVERALL *(continued)*

8. Has your child had any medical problems in the last several months? If yes, explain: ◯ YES ◯ NO

9. Do you have any concerns about your child's behavior? If yes, explain: ◯ YES ◯ NO

10. Does anything about your child worry you? If yes, explain: ◯ YES ◯ NO

Ages & Stages Questionnaires®, Third Edition (ASQ-3™), Squires & Bricker
© 2009 Paul H. Brookes Publishing Co. All rights reserved.

48 Month ASQ-3 Information Summary

45 months 0 days through 50 months 30 days

Child's name: ______________________ Date ASQ completed: ______________________

Child's ID #: ______________________ Date of birth: ______________________

Administering program/provider: ______________________

1. **SCORE AND TRANSFER TOTALS TO CHART BELOW:** See *ASQ-3 User's Guide* for details, including how to adjust scores if item responses are missing. Score each item (YES = 10, SOMETIMES = 5, NOT YET = 0). Add item scores, and record each area total. In the chart below, transfer the total scores, and fill in the circles corresponding with the total scores.

Area	Cutoff	Total Score	0	5	10	15	20	25	30	35	40	45	50	55	60
Communication	30.72		●	●	●	●	●	●	●	○	○	○	○	○	○
Gross Motor	32.78		●	●	●	●	●	●	●	○	○	○	○	○	○
Fine Motor	15.81		●	●	●	●	○	○	○	○	○	○	○	○	○
Problem Solving	31.30		●	●	●	●	●	●	●	○	○	○	○	○	○
Personal-Social	26.60		●	●	●	●	●	●	○	○	○	○	○	○	○

2. **TRANSFER OVERALL RESPONSES:** Bolded uppercase responses require follow-up. See *ASQ-3 User's Guide*, Chapter 6.

1. Hears well? Yes **NO**
   Comments:
2. Talks like other children his age? Yes **NO**
   Comments:
3. Understand most of what your child says? Yes **NO**
   Comments:
4. Others understand most of what your child says? Yes **NO**
   Comments:
5. Walks, runs, and climbs like other children? Yes **NO**
   Comments:
6. Family history of hearing impairment? **YES** No
   Comments:
7. Concerns about vision? **YES** No
   Comments:
8. Any medical problems? **YES** No
   Comments:
9. Concerns about behavior? **YES** No
   Comments:
10. Other concerns? **YES** No
    Comments:

3. **ASQ SCORE INTERPRETATION AND RECOMMENDATION FOR FOLLOW-UP:** You must consider total area scores, overall responses, and other considerations, such as opportunities to practice skills, to determine appropriate follow-up.

If the child's total score is in the ☐ area, it is above the cutoff, and the child's development appears to be on schedule.
If the child's total score is in the ▒ area, it is close to the cutoff. Provide learning activities and monitor.
If the child's total score is in the ■ area, it is below the cutoff. Further assessment with a professional may be needed.

4. **FOLLOW-UP ACTION TAKEN:** Check all that apply.

_____ Provide activities and rescreen in _____ months.
_____ Share results with primary health care provider.
_____ Refer for (circle all that apply) hearing, vision, and/or behavioral screening.
_____ Refer to primary health care provider or other community agency (specify reason): ______________________.
_____ Refer to early intervention/early childhood special education.
_____ No further action taken at this time
_____ Other (specify): ______________________

5. **OPTIONAL:** Transfer item responses (Y = YES, S = SOMETIMES, N = NOT YET, X = response missing).

	1	2	3	4	5	6
Communication						
Gross Motor						
Fine Motor						
Problem Solving						
Personal-Social						

P101480800

Ages & Stages Questionnaires®, Third Edition (ASQ-3™), Squires & Bricker
© 2009 Paul H. Brookes Publishing Co. All rights reserved.
