# Supplementary material for: Neurodevelopmental outcome in children between one and five years after persistent pulmonary hypertension of term and near-term newborns
Source: Front Pediatr. 2024 Oct 23;12:1450916. doi: 10.3389/fped.2024.1450916 (PMC11538055; doi:10.3389/fped.2024.1450916)
Supplement: Supplementary file 6 [file Datasheet4.pdf]

ASQ-3™ Ages & Stages Questionnaires®

# 60 Month Questionnaire

*57 months 0 days through 66 months 0 days*

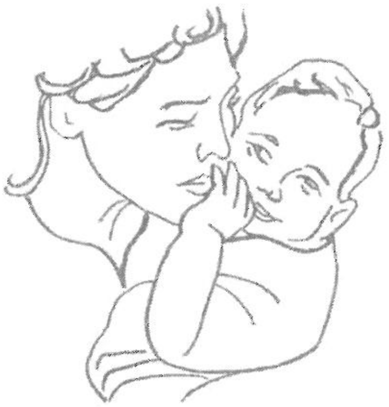

Please provide the following information. Use black or blue ink only and print legibly when completing this form.

Date ASQ completed: M M D D Y Y Y Y

## Child's information

Child's first name:

Middle initial:

Child's last name:

Child's date of birth: M M D D Y Y Y Y

Child's gender:
- ◯ Male
- ◯ Female

## Person filling out questionnaire

First name:

Middle initial:

Last name:

Street address:

Relationship to child:
- ◯ Parent
- ◯ Guardian
- ◯ Teacher
- ◯ Child care provider
- ◯ Grandparent or other relative
- ◯ Foster parent
- ◯ Other:

City:

State/Province:

ZIP/Postal code:

Country:

Home telephone number:

Other telephone number:

E-mail address:

Names of people assisting in questionnaire completion:

## PROGRAM INFORMATION

Child ID #:

Program ID #:

Program name:

E101600101

*Ages & Stages Questionnaires®, Third Edition (ASQ-3™)*, Squires & Bricker
© 2009 Paul H. Brookes Publishing Co. All rights reserved.

ASQ-3™

# 60 Month Questionnaire

57 months 0 days through 66 months 0 days

On the following pages are questions about activities children may do. Your child may have already done some of the activities described here, and there may be some your child has not begun doing yet. For each item, please fill in the circle that indicates whether your child is doing the activity regularly, sometimes, or not yet.

**Important Points to Remember:**

- ☑ Try each activity with your child before marking a response.
- ☑ Make completing this questionnaire a game that is fun for you and your child.
- ☑ Make sure your child is rested and fed.
- ☑ Please return this questionnaire by ______________.

**Notes:**

______________________________

______________________________

______________________________

______________________________

## COMMUNICATION

| | YES | SOMETIMES | NOT YET | |
|---|---|---|---|---|
| 1. Without your giving help by pointing or repeating directions, does your child follow three directions that are *unrelated* to one another? Give all three directions before your child starts. For example, you may ask your child, "Clap your hands, walk to the door, and sit down," or "Give me the pen, open the book, and stand up." | ○ | ○ | ○ | ___ |
| 2. Does your child use four- and five-word sentences? For example, does your child say, "I want the car"? Please write an example: | ○ | ○ | ○ | ___ |
| 3. When talking about something that already happened, does your child use words that end in "-ed," such as "walk*ed*," "jump*ed*," or "play*ed*"? Ask your child questions, such as "How did you get to the store?" ("We walk*ed*.") "What did you do at your friend's house?" ("We play*ed*.") Please write an example: | ○ | ○ | ○ | ___ |
| 4. Does your child use comparison words, such as "heavier," "stronger," or "shorter"? Ask your child questions, such as "A car is big, but a bus is _____" (bigger); "A cat is heavy, but a man is _____" (heavier); "A TV is small, but a book is _____" (smaller). Please write an example: | ○ | ○ | ○ | ___ |



E101600201

*Ages & Stages Questionnaires®, Third Edition (ASQ-3™)*, Squires & Bricker
© 2009 Paul H. Brookes Publishing Co. All rights reserved.

## COMMUNICATION *(continued)*

| | YES | SOMETIMES | NOT YET | |
|---|---|---|---|---|
| 5. Does your child answer the following questions? *(Mark "sometimes" if your child answers only one question.)* | ○ | ○ | ○ | ___ |

"What do you do when you are hungry?" *(Acceptable answers include "get food," "eat," "ask for something to eat," and "have a snack.")* Please write your child's response:

"What do you do when you are tired?" *(Acceptable answers include: "take a nap," "rest," "go to sleep," "go to bed," "lie down," and "sit down.")* Please write your child's response:

| | YES | SOMETIMES | NOT YET | |
|---|---|---|---|---|
| 6. Does your child repeat the sentences shown below back to you, without any mistakes? *(Read the sentences one at a time. You may repeat each sentence one time. Mark "yes" if your child repeats both sentences without mistakes or "sometimes" if your child repeats one sentence without mistakes.)* | ○ | ○ | ○ | ___ |

Jane hides her shoes for Maria to find.

Al read the blue book under his bed.

COMMUNICATION TOTAL ___

## GROSS MOTOR

| | YES | SOMETIMES | NOT YET | |
|---|---|---|---|---|
| 1. While standing, does your child throw a ball *overhand* in the direction of a person standing at least 6 feet away? To throw overhand, your child must raise his arm to shoulder height and throw the ball forward. *(Dropping the ball or throwing the ball underhand should be scored as "not yet.")* | ○ | ○ | ○ | ___ |

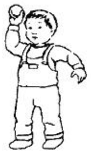

| | YES | SOMETIMES | NOT YET | |
|---|---|---|---|---|
| 2. Does your child catch a large ball with both hands? *(You should stand about 5 feet away and give your child two or three tries before you mark the answer.)* | ○ | ○ | ○ | ___ |

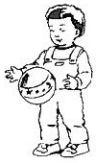

| | YES | SOMETIMES | NOT YET | |
|---|---|---|---|---|
| 3. Without holding onto anything, does your child stand on one foot for at least 5 seconds without losing her balance and putting her foot down? *(You may give your child two or three tries before you mark the answer.)* | ○ | ○ | ○ | ___ |

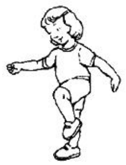



*Ages & Stages Questionnaires®, Third Edition (ASQ-3™), Squires & Bricker*
© 2009 Paul H. Brookes Publishing Co. All rights reserved.

## GROSS MOTOR *(continued)*

| | YES | SOMETIMES | NOT YET | |
|---|---|---|---|---|
| 4. Does your child walk on his tiptoes for 15 feet (about the length of a large car)? *(You may show him how to do this.)* | ○ | ○ | ○ | ___ |
| 5. Does your child hop forward on one foot for a distance of 4–6 feet without putting down the other foot? *(You may give her two tries on each foot. Mark "sometimes" if she can hop on one foot only.)* | ○ | ○ | ○ | ___ |
| 6. Does your child skip using alternating feet? *(You may show him how to do this.)* | ○ | ○ | ○ | ___ |

GROSS MOTOR TOTAL ___

## FINE MOTOR

| | YES | SOMETIMES | NOT YET | |
|---|---|---|---|---|
| 1. Ask your child to trace on the line below with a pencil. Does your child trace on the line without going off the line more than two times? *(Mark "sometimes" if your child goes off the line three times.)* | ○ | ○ | ○ | ___ |
| 2. Ask your child to draw a picture of a person on a blank sheet of paper. You may ask your child, "Draw a picture of a girl or a boy." If your child draws a person with head, body, arms, *and* legs, mark "yes." If your child draws a person with only three parts (head, body, arms, or legs), mark "sometimes." If your child draws a person with two or fewer parts (head, body, arms, or legs), mark "not yet." Be sure to include the sheet of paper with your child's drawing with this questionnaire. | ○ | ○ | ○ | ___ |
| 3. Draw a line across a piece of paper. Using child-safe scissors, does your child cut the paper in half on a more or less straight line, making the blades go up and down? *(Carefully watch your child's use of scissors for safety reasons.)* | ○ | ○ | ○ | ___ |
| 4. Using the shapes below to look at, does your child copy the shapes in the space below without tracing? *(Your child's drawings should look similar to the design of the shapes below, but they may be different in size. Mark "yes" if she copies all three shapes; mark "sometimes" if your child copies two shapes.)* | ○ | ○ | ○ | ___ |

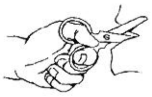

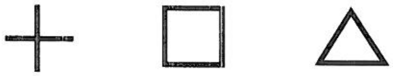

(Space for child's shapes)



*Ages & Stages Questionnaires®, Third Edition (ASQ-3™)*, Squires & Bricker
© 2009 Paul H. Brookes Publishing Co. All rights reserved.

## FINE MOTOR *(continued)*

| | YES | SOMETIMES | NOT YET | |
|---|---|---|---|---|
| 5. Using the letters below to look at, does your child copy the letters without tracing? Cover up all of the letters except the letter being copied. *(Mark "yes" if your child copies four of the letters and you can read them. Mark "sometimes" if your child copies two or three letters and you can read them.)* | ○ | ○ | ○ | ___ |

V H T C A

(Space for child's letters)

| | YES | SOMETIMES | NOT YET | |
|---|---|---|---|---|
| 6. Print your child's first name. Can your child copy the letters? The letters may be large, backward, or reversed. *(Mark "sometimes" if your child copies about half of the letters.)* | ○ | ○ | ○ | ___ |

(Space for adult's printing)

(Space for child's printing)

FINE MOTOR TOTAL ___

## PROBLEM SOLVING

| | YES | SOMETIMES | NOT YET | |
|---|---|---|---|---|
| 1. When asked, "Which circle is smallest?" does your child point to the smallest circle? *(Ask this question without providing help by pointing, gesturing, or looking at the smallest circle.)* | ○ | ○ | ○ | ___ |

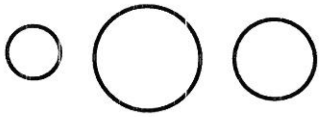

| | YES | SOMETIMES | NOT YET | |
|---|---|---|---|---|
| 2. When shown objects and asked, "What color is this?" does your child name five different colors like red, blue, yellow, orange, black, white, or pink? *(Mark "yes" only if your child answers the question correctly using five colors.)* | ○ | ○ | ○ | ___ |



*Ages & Stages Questionnaires®, Third Edition (ASQ-3™)*, Squires & Bricker
© 2009 Paul H. Brookes Publishing Co. All rights reserved.

## PROBLEM SOLVING *(continued)*

| | YES | SOMETIMES | NOT YET | |
|---|---|---|---|---|
| 3. Does your child count up to 15 without making mistakes? If so, mark "yes." If your child counts to 12 without making mistakes, mark "sometimes." | ○ | ○ | ○ | ___ |
| 4. Does your child finish the following sentences using a word that means the opposite of the word that is italicized? For example: "A rock is *hard*, and a pillow is *soft*." | ○ | ○ | ○ | ___ |

Please write your child's responses below:

A cow is *big*, and a mouse is [ ]

Ice is *cold*, and fire is [ ]

We see stars at *night*, and we see the sun during the [ ]

When I throw the ball *up*, it comes [ ]

*(Mark "yes" if he finishes three of four sentences correctly. Mark "sometimes" if he finishes two of four sentences correctly.)*

| | YES | SOMETIMES | NOT YET | |
|---|---|---|---|---|
| 5. Does your child know the names of numbers? *(Mark "yes" if she identifies the three numbers below. Mark "sometimes" if she identifies two numbers.)* | ○ | ○ | ○ | ___ |

**3 1 2**

| | YES | SOMETIMES | NOT YET | |
|---|---|---|---|---|
| 6. Does your child name at least four letters in her name? Point to the letters and ask, "What letter is this?" *(Point to the letters out of order.)* | ○ | ○ | ○ | ___ |

PROBLEM SOLVING TOTAL ___

## PERSONAL-SOCIAL

| | YES | SOMETIMES | NOT YET | |
|---|---|---|---|---|
| 1. Can your child serve himself, taking food from one container to another, using utensils? For example, does your child use a large spoon to scoop applesauce from a jar into a bowl? | ○ | ○ | ○ | ___ |
| 2. Does your child wash her hands and face using soap and water and dry off with a towel without help? | ○ | ○ | ○ | ___ |
| 3. Does your child tell you at least four of the following? Please mark the items your child knows. | ○ | ○ | ○ | ___ |

○ a. First name ○ d. Last name

○ b. Age ○ e. Boy or girl

○ c. City he lives in ○ f. Telephone number



*Ages & Stages Questionnaires®, Third Edition (ASQ-3™)*, Squires & Bricker
© 2009 Paul H. Brookes Publishing Co. All rights reserved.

## PERSONAL-SOCIAL *(continued)*

| | YES | SOMETIMES | NOT YET | |
|---|---|---|---|---|
| 4. Does your child dress and undress himself, including buttoning medium-size buttons and zipping front zippers? | ○ | ○ | ○ | ___ |
| 5. Does your child use the toilet by herself? *(She goes to the bathroom, sits on the toilet, wipes, and flushes.)* Mark "yes" even if she does this after you remind her. | ○ | ○ | ○ | ___ |
| 6. Does your child usually take turns and share with other children? | ○ | ○ | ○ | ___ |

PERSONAL-SOCIAL TOTAL ___

## OVERALL

*Parents and providers may use the space below for additional comments.*

1. Do you think your child hears well? If no, explain: ○ YES ○ NO

2. Do you think your child talks like other children her age? If no, explain: ○ YES ○ NO

3. Can you understand most of what your child says? If no, explain: ○ YES ○ NO

4. Can other people understand most of what your child says? If no, explain: ○ YES ○ NO



*Ages & Stages Questionnaires®, Third Edition (ASQ-3™)*, Squires & Bricker
© 2009 Paul H. Brookes Publishing Co. All rights reserved.

## OVERALL *(continued)*

5. Do you think your child walks, runs, and climbs like other children his age? If no, explain: ○ YES ○ NO

6. Does either parent have a family history of childhood deafness or hearing impairment? If yes, explain: ○ YES ○ NO

7. Do you have any concerns about your child's vision? If yes, explain: ○ YES ○ NO

8. Has your child had any medical problems in the last several months? If yes, explain: ○ YES ○ NO

9. Do you have any concerns about your child's behavior? If yes, explain: ○ YES ○ NO

10. Does anything about your child worry you? If yes, explain: ○ YES ○ NO



*Ages & Stages Questionnaires®, Third Edition (ASQ-3™)*, Squires & Bricker
© 2009 Paul H. Brookes Publishing Co. All rights reserved.

# 60 Month ASQ-3 Information Summary

*57 months 0 days through 66 months 0 days*

Child's name: ______________________ Date ASQ completed: ______________________

Child's ID #: ______________________ Date of birth: ______________________

Administering program/provider: ______________________

1. **SCORE AND TRANSFER TOTALS TO CHART BELOW:** See *ASQ-3 User's Guide* for details, including how to adjust scores if item responses are missing. Score each item (YES = 10, SOMETIMES = 5, NOT YET = 0). Add item scores, and record each area total. In the chart below, transfer the total scores, and fill in the circles corresponding with the total scores.

| Area | Cutoff | Total Score | 0 | 5 | 10 | 15 | 20 | 25 | 30 | 35 | 40 | 45 | 50 | 55 | 60 |
|---|---|---|---|---|---|---|---|---|---|---|---|---|---|---|---|
| Communication | 33.19 | | | | | | | | | | | | | | |
| Gross Motor | 31.28 | | | | | | | | | | | | | | |
| Fine Motor | 26.54 | | | | | | | | | | | | | | |
| Problem Solving | 29.99 | | | | | | | | | | | | | | |
| Personal-Social | 39.07 | | | | | | | | | | | | | | |

2. **TRANSFER OVERALL RESPONSES:** Bolded uppercase responses require follow-up. See *ASQ-3 User's Guide*, Chapter 6.

1. Hears well? Yes **NO**
   Comments:
2. Talks like other children his age? Yes **NO**
   Comments:
3. Understand most of what your child says? Yes **NO**
   Comments:
4. Others understand most of what your child says? Yes **NO**
   Comments:
5. Walks, runs, and climbs like other children? Yes **NO**
   Comments:
6. Family history of hearing impairment? **YES** No
   Comments:
7. Concerns about vision? **YES** No
   Comments:
8. Any medical problems? **YES** No
   Comments:
9. Concerns about behavior? **YES** No
   Comments:
10. Other concerns? **YES** No
    Comments:

3. **ASQ SCORE INTERPRETATION AND RECOMMENDATION FOR FOLLOW-UP:** You must consider total area scores, overall responses, and other considerations, such as opportunities to practice skills, to determine appropriate follow-up.

If the child's total score is in the ☐ (white) area, it is above the cutoff, and the child's development appears to be on schedule.
If the child's total score is in the (grey) area, it is close to the cutoff. Provide learning activities and monitor.
If the child's total score is in the ■ (black) area, it is below the cutoff. Further assessment with a professional may be needed.

4. **FOLLOW-UP ACTION TAKEN:** Check all that apply.

_____ Provide activities and rescreen in _____ months.

_____ Share results with primary health care provider.

_____ Refer for (circle all that apply) hearing, vision, and/or behavioral screening.

_____ Refer to primary health care provider or other community agency (specify reason): ______________________.

_____ Refer to early intervention/early childhood special education.

_____ No further action taken at this time

_____ Other (specify): ______________________

5. **OPTIONAL:** Transfer item responses (Y = YES, S = SOMETIMES, N = NOT YET, X = response missing).

| | 1 | 2 | 3 | 4 | 5 | 6 |
|---|---|---|---|---|---|---|
| Communication | | | | | | |
| Gross Motor | | | | | | |
| Fine Motor | | | | | | |
| Problem Solving | | | | | | |
| Personal-Social | | | | | | |

P101600901

*Ages & Stages Questionnaires®, Third Edition (ASQ-3™)*, Squires & Bricker
© 2009 Paul H. Brookes Publishing Co. All rights reserved.
