# Supplementary material for: Neurodevelopmental outcome in children between one and five years after persistent pulmonary hypertension of term and near-term newborns
Source: Front Pediatr. 2024 Oct 23;12:1450916. doi: 10.3389/fped.2024.1450916 (PMC11538055; doi:10.3389/fped.2024.1450916)
Supplement: Supplementary file 7 [file Datasheet5.pdf]

ASQ-3™ Ages & Stages Questionnaires®

36 Month Questionnaire

34 months 16 days through 38 months 30 days

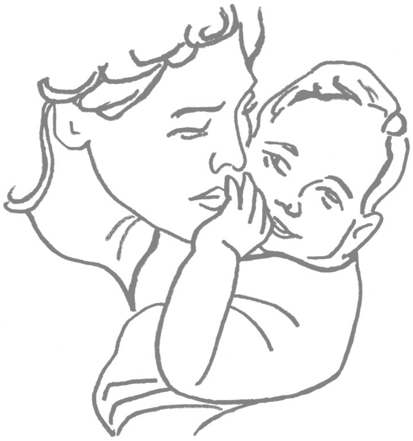

Please provide the following information. Use black or blue ink only and print legibly when completing this form.

Date ASQ completed: ____________________

Child's information

Child's first name: ____________ Middle initial: ____ Child's last name: ____________

Child's date of birth: ____________________

Child's gender:
◯ Male ◯ Female

Person filling out questionnaire

First name: ____________ Middle initial: ____ Last name: ____________

Street address: ____________________

Relationship to child:
◯ Parent ◯ Guardian ◯ Teacher ◯ Child care provider
◯ Grandparent or other relative ◯ Foster parent ◯ Other: ____________

City: ____________ State/Province: ____________ ZIP/Postal code: ____________

Country: ____________ Home telephone number: ____________ Other telephone number: ____________

E-mail address: ____________________

Names of people assisting in questionnaire completion: ____________________

____

Program Information

Child ID #: ____________________

Program ID #: ____________________

Program name: ____________________

P101360100

Ages & Stages Questionnaires®, Third Edition (ASQ-3™), Squires & Bricker
© 2009 Paul H. Brookes Publishing Co. All rights reserved.

ASQ-3™

36 Month Questionnaire

34 months 16 days through 38 months 30 days

On the following pages are questions about activities children may do. Your child may have already done some of the activities described here, and there may be some your child has not begun doing yet. For each item, please fill in the circle that indicates whether your child is doing the activity regularly, sometimes, or not yet.

Important Points to Remember:

- ☑ Try each activity with your child before marking a response.
- ☑ Make completing this questionnaire a game that is fun for you and your child.
- ☑ Make sure your child is rested and fed.
- ☑ Please return this questionnaire by ______________.

Notes:

____

____

____

____

COMMUNICATION

	YES	SOMETIMES	NOT YET	
1. When you ask your child to point to her nose, eyes, hair, feet, ears, and so forth, does she correctly point to at least *seven* body parts? *(She can point to parts of herself, you, or a doll. Mark "sometimes" if she correctly points to at least three different body parts.)*	○	○	○	___
2. Does your child make sentences that are three or four words long? Please give an example:	○	○	○	___
3. Without giving your child help by pointing or using gestures, ask him to "put the book *on* the table" and "put the shoe *under* the chair." Does your child carry out both of these directions correctly?	○	○	○	___
4. When looking at a picture book, does your child tell you what is happening or what action is taking place in the picture (for example, "barking," "running," "eating," or "crying")? You may ask, "What is the dog (or boy) doing?"	○	○	○	___
5. Show your child how a zipper on a coat moves up and down, and say, "See, this goes up and down." Put the zipper to the middle and ask your child to move the zipper *down*. Return the zipper to the middle and ask your child to move the zipper *up*. Do this several times, placing the zipper in the middle before asking your child to move it up or down. Does your child consistently move the zipper up when you say "up" and down when you say "down"?	○	○	○	___
6. When you ask, "What is your name?" does your child say both her first and last names?	○	○	○	___

COMMUNICATION TOTAL ___

E101360200

Ages & Stages Questionnaires®, Third Edition (ASQ-3™), Squires & Bricker
© 2009 Paul H. Brookes Publishing Co. All rights reserved.

GROSS MOTOR

YES | SOMETIMES | NOT YET

1. Without holding onto anything for support, does your child kick a ball by swinging his leg forward? ○ ○ ○ ___

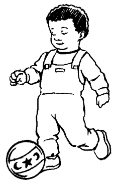

2. Does your child jump with both feet leaving the floor at the same time? ○ ○ ○ ___

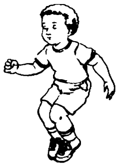

3. Does your child walk up stairs, using only one foot on each stair? *(The left foot is on one step, and the right foot is on the next.)* She may hold onto the railing or wall. *(You can look for this at a store, on a playground, or at home.)* ○ ○ ○ ___

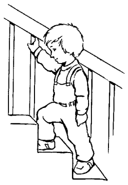

4. Does your child stand on one foot for about 1 second without holding onto anything? ○ ○ ○ ___

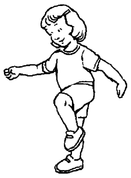

5. While standing, does your child throw a ball *overhand* by raising his arm to shoulder height and throwing the ball forward? *(Dropping the ball or throwing the ball underhand should be scored as "not yet.")* ○ ○ ○ ___

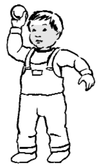

6. Does your child jump forward at least 6 inches with both feet leaving the ground at the same time? ○ ○ ○ ___

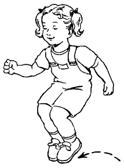

GROSS MOTOR TOTAL ___

FINE MOTOR

YES | SOMETIMES | NOT YET

1. After your child watches you draw a line from the top of the paper to the bottom with a pencil, crayon, or pen, ask her to make a line like yours. Do not let your child trace your line. Does your child copy you by drawing a single line in a vertical direction? ○ ○ ○ ___

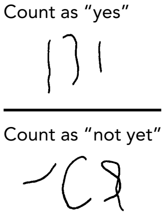

E101360300

Ages & Stages Questionnaires®, Third Edition (ASQ-3™), Squires & Bricker
© 2009 Paul H. Brookes Publishing Co. All rights reserved.

FINE MOTOR *(continued)*

	YES	SOMETIMES	NOT YET	
2. Can your child string small items such as beads, macaroni, or pasta "wagon wheels" onto a string or shoelace? 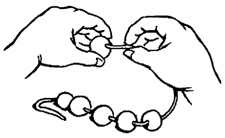	○	○	○	___
3. After your child watches you draw a single circle, ask him to make a circle like yours. Do not let him trace your circle. Does your child copy you by drawing a circle? Count as "yes" 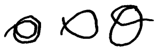 Count as "not yet" 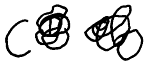	○	○	○	___
4. After your child watches you draw a line from one side of the paper to the other side, ask her to make a line like yours. Do not let your child trace your line. Does your child copy you by drawing a single line in a horizontal direction? Count as "yes" 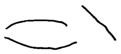 Count as "not yet" 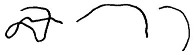	○	○	○	___
5. Does your child try to cut paper with child-safe scissors? He does not need to cut the paper but must get the blades to open and close while holding the paper with the other hand. *(You may show your child how to use scissors. Carefully watch your child's use of scissors for safety reasons.)* 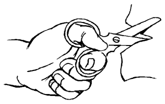	○	○	○	___
6. When drawing, does your child hold a pencil, crayon, or pen between her fingers and thumb like an adult does?	○	○	○	___

FINE MOTOR TOTAL ___

PROBLEM SOLVING

	YES	SOMETIMES	NOT YET	
1. While your child watches, line up four objects like blocks or cars in a row. Does your child copy or imitate you and line up *four* objects in a row? *(You can also use spools of thread, small boxes, or other toys.)* 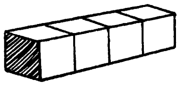	○	○	○	___
2. If your child wants something he cannot reach, does he find a chair or box to stand on to reach it (for example, to get a toy on a counter or to "help" you in the kitchen)?	○	○	○	___

Ages & Stages Questionnaires®, Third Edition (ASQ-3™), Squires & Bricker
© 2009 Paul H. Brookes Publishing Co. All rights reserved.

PROBLEM SOLVING *(continued)*

	YES	SOMETIMES	NOT YET	
3. When you point to the figure and ask your child, "What is this?" does your child say a word that means a person or something similar? *(Mark "yes" for responses like "snowman," "boy," "man," "girl," "Daddy," "spaceman," and "monkey.")* Please write your child's response here:	○	○	○	___

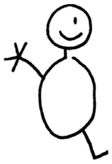

	YES	SOMETIMES	NOT YET	
4. When you say, "Say 'seven three,'" does your child repeat *just* the two numbers in the same order? ***Do not repeat the numbers.*** If necessary, try another pair of numbers and say, "Say 'eight two.'" *(Your child must repeat just one series of two numbers for you to answer "yes" to this question.)*	○	○	○	___
5. Show your child how to make a bridge with blocks, boxes, or cans, like the example. Does your child copy you by making one like it?	○	○	○	___
6. When you say, "Say 'five eight three,'" does your child repeat *just* the three numbers in the same order? ***Do not repeat the numbers.*** If necessary, try another series of numbers and say, "Say 'six nine two.'" *(Your child must repeat just one series of three numbers for you to answer "yes" to this question.)*	○	○	○	___

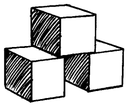

PROBLEM SOLVING TOTAL ___

PERSONAL-SOCIAL

	YES	SOMETIMES	NOT YET	
1. Does your child use a spoon to feed herself with little spilling?	○	○	○	___
2. Does your child push a little wagon, stroller, or toy on wheels, steering it around objects and backing out of corners if he cannot turn?	○	○	○	___
3. When your child is looking in a mirror and you ask, "Who is in the mirror?" does she say either "me" or her own name?	○	○	○	___
4. Does your child put on a coat, jacket, or shirt by himself?	○	○	○	___
5. Using these exact words, ask your child, "Are you a girl or a boy?" Does your child answer correctly?	○	○	○	___
6. Does your child take turns by waiting while another child or adult takes a turn?	○	○	○	___

PERSONAL-SOCIAL TOTAL ___

Ages & Stages Questionnaires®, Third Edition (ASQ-3™), Squires & Bricker
© 2009 Paul H. Brookes Publishing Co. All rights reserved.

OVERALL

Parents and providers may use the space below for additional comments.

1. Do you think your child hears well? If no, explain: ○ YES ○ NO

2. Do you think your child talks like other children her age? If no, explain: ○ YES ○ NO

3. Can you understand most of what your child says? If no, explain: ○ YES ○ NO

4. Can other people understand most of what your child says? If no, explain: ○ YES ○ NO

5. Do you think your child walks, runs, and climbs like other children his age? If no, explain: ○ YES ○ NO

6. Does either parent have a family history of childhood deafness or hearing impairment? If yes, explain: ○ YES ○ NO

Ages & Stages Questionnaires®, Third Edition (ASQ-3™), Squires & Bricker
© 2009 Paul H. Brookes Publishing Co. All rights reserved.

OVERALL *(continued)*

7. Do you have any concerns about your child's vision? If yes, explain: ○ YES ○ NO

8. Has your child had any medical problems in the last several months? If yes, explain: ○ YES ○ NO

9. Do you have any concerns about your child's behavior? If yes, explain: ○ YES ○ NO

10. Does anything about your child worry you? If yes, explain: ○ YES ○ NO

Ages & Stages Questionnaires®, Third Edition (ASQ-3™), Squires & Bricker
© 2009 Paul H. Brookes Publishing Co. All rights reserved.

36 Month ASQ-3 Information Summary

34 months 16 days through 38 months 30 days

Child's name: ______________________________ Date ASQ completed: ______________________________

Child's ID #: ______________________________ Date of birth: ______________________________

Administering program/provider: ______________________________

1. **SCORE AND TRANSFER TOTALS TO CHART BELOW:** See *ASQ-3 User's Guide* for details, including how to adjust scores if item responses are missing. Score each item (YES = 10, SOMETIMES = 5, NOT YET = 0). Add item scores, and record each area total. In the chart below, transfer the total scores, and fill in the circles corresponding with the total scores.

Area	Cutoff	Total Score	0	5	10	15	20	25	30	35	40	45	50	55	60
Communication	30.99		●	●	●	●	●	●	●	○	○	○	○	○	○
Gross Motor	36.99		●	●	●	●	●	●	●	●	○	○	○	○	○
Fine Motor	18.07		●	●	●	●	○	○	○	○	○	○	○	○	○
Problem Solving	30.29		●	●	●	●	●	●	●	○	○	○	○	○	○
Personal-Social	35.33		●	●	●	●	●	●	●	●	○	○	○	○	○

2. **TRANSFER OVERALL RESPONSES:** Bolded uppercase responses require follow-up. See *ASQ-3 User's Guide,* Chapter 6.

1. Hears well? Yes **NO**
   Comments:
2. Talks like other children his age? Yes **NO**
   Comments:
3. Understand most of what your child says? Yes **NO**
   Comments:
4. Others understand most of what your child says? Yes **NO**
   Comments:
5. Walks, runs, and climbs like other children? Yes **NO**
   Comments:
6. Family history of hearing impairment? **YES** No
   Comments:
7. Concerns about vision? **YES** No
   Comments:
8. Any medical problems? **YES** No
   Comments:
9. Concerns about behavior? **YES** No
   Comments:
10. Other concerns? **YES** No
    Comments:

3. **ASQ SCORE INTERPRETATION AND RECOMMENDATION FOR FOLLOW-UP:** You must consider total area scores, overall responses, and other considerations, such as opportunities to practice skills, to determine appropriate follow-up.

If the child's total score is in the ☐ area, it is above the cutoff, and the child's development appears to be on schedule.
If the child's total score is in the ▒ area, it is close to the cutoff. Provide learning activities and monitor.
If the child's total score is in the ■ area, it is below the cutoff. Further assessment with a professional may be needed.

4. **FOLLOW-UP ACTION TAKEN:** Check all that apply.

______ Provide activities and rescreen in _____ months.

______ Share results with primary health care provider.

______ Refer for (circle all that apply) hearing, vision, and/or behavioral screening.

______ Refer to primary health care provider or other community agency (specify reason): ______________________________.

______ Refer to early intervention/early childhood special education.

______ No further action taken at this time

______ Other (specify): ______________________________

5. **OPTIONAL:** Transfer item responses (Y = YES, S = SOMETIMES, N = NOT YET, X = response missing).

	1	2	3	4	5	6
Communication						
Gross Motor						
Fine Motor						
Problem Solving						
Personal-Social						

P101360800

Ages & Stages Questionnaires®, Third Edition (ASQ-3™), Squires & Bricker
© 2009 Paul H. Brookes Publishing Co. All rights reserved.
